# Comparisons of exacerbations and mortality among LAMA/LABA combinations in stable chronic obstructive pulmonary disease: systematic review and Bayesian network meta-analysis

**DOI:** 10.1186/s12931-020-01540-8

**Published:** 2020-11-25

**Authors:** Hyun Woo Lee, Jimyung Park, Eun Jin Jang, Chang-Hoon Lee

**Affiliations:** 1 Division of Pulmonary and Critical Care, Department of Internal Medicine, Seoul Metropolitan Government-Seoul National University Boramae Medical Center, 20 Boramae-ro-5-gil, Dongjak-gu, Seoul, 07061 South Korea; 2 Division of Pulmonary and Critical Care Medicine, Department of Internal Medicine, Seoul National University Hospital, 101 Daehak-Ro Jongno-Gu, Seoul, 03080 Republic of Korea; 3 Department of Information Statistics, Andong National University, 1375 Gyeongdong-ro, Andong si, 760749 South Korea

**Keywords:** Pulmonary disease, Chronic obstructive, Respiratory therapy, Administration, Inhalation, Symptom flare-up, Mortality, Drug-related side effects and adverse reactions, Bayes theorem, Network meta-analysis

## Abstract

**Background:**

Only few randomized controlled trials (RCTs) for head-to-head comparison have been conducted between various combinations of long-acting muscarinic antagonists (LAMAs) and long-acting beta-agonists (LABAs). Our study was conducted to compare acute exacerbation and all-cause mortality among different LAMA/LABA regimens using Bayesian network meta-analysis (NMA).

**Methods:**

We searched Medline, EMBASE, and the Cochrane library (search date: July 1, 2019). We included parallel-group RCTs comparing LAMA/LABA combinations with other inhaled drugs in the stable COPD for ≥ 48 weeks. Two different network geometries were used. The geometry of network (A) had nodes of individual drugs or their combination, while that of network (B) combined all other treatments except LAMA/LABA into each drug class. This study was prospectively registered in PROSPERO; CRD42019126753.

**Results:**

We included 16 RCTs involving a total of 39,065 patients with stable COPD. Six combinations of LAMA/LABA were identified: tiotropium/salmeterol, glycopyrrolate/indacaterol, umeclidinium/vilanterol, tiotropium/olodaterol, aclidinium/formoterol, and glycopyrrolate/formoterol. We found that umeclidinium/vilanterol was associated with a lower risk of total exacerbations than other LAMA/LABAs in the NMA using network (A) (level of evidence: low or moderate). However, the significant differences were not present in the NMA of network (B). There were no significant differences among the LAMA/LABA combinations in terms of the number of moderate to severe exacerbations, all-cause mortality, major adverse cardiovascular events, or pneumonia.

**Conclusions:**

The present NMA including all available RCTs provided that there is no strong evidence suggesting different benefits among LAMA/LABAs in patients with stable COPD who have been followed up for 48 weeks or more.

*Trial registration*: This study was prospectively registered in PROSPERO; CRD42019126753.

## Background

Long acting bronchodilators such as long-acting muscarinic antagonists (LAMAs) and long-acting beta-agonists (LABAs) are the main drugs used to treat stable chronic obstructive pulmonary disease (COPD). They improve symptoms and exercise performance status by reducing small airway limitations, hyperinflation [1, 2], and exacerbation risk [3–5]. Recently, LAMA/LABA combination therapy has been introduced as a more potent treatment than LAMA or LABA alone. Studies and meta-analyses have shown that such combination therapy has a greater effect than monotherapy on lung function, symptoms, quality of life, and acute exacerbations [6–14]. In addition, LAMA/LABA combination therapy is associated with less frequent moderate to severe exacerbations than inhaled corticosteroid (ICS)/LABA combination [8, 9, 15], although a large trial contradicted these results [16]. Currently, in patients with stable COPD whose symptoms or exacerbations cannot be controlled using a single long-acting bronchodilator, LAMA/LABA combination therapy is primarily recommended [17].

A variety of LAMA/LABA combinations are available on the market, but it is not clear which are the best choice; all LAMA/LABA therapies are expected to have similar efficacy [10, 18], even though only a few head-to-head trials have been carried out with a relatively short study duration [19–23]. Instead, several efforts have been made to indirectly compare the efficacy of the LAMA/LABA combinations [24, 25], although no previous studies have compared exacerbation risk or mortality risk, which are the two most important outcomes in patients with stable COPD. Furthermore, no studies have taken into consideration the outcomes of recently conducted large trials [15, 16, 26]. The present systematic review (SR) and Bayesian network meta-analysis (NMA) included all available long-term trials and aimed to compare efficacy and safety, comparing the risk of acute exacerbation and mortality between different LAMA/LABA combinations.

## Methods

### Protocol and registration

We followed the guidelines of the Preferred Reporting Items for SRs and Meta-analyses extension statement, which incorporates NMAs as medical interventions [27], as well as the BayesWatch guidelines for reporting estimated results using Bayesian methods [28]. We registered our study protocol in the International Prospective Register of Systematic Reviews (PROSPERO, CRD42019126753).

### Eligibility criteria

We included clinical studies that met the following eligibility criteria: (1) adult patients with stable COPD; (2) treatment with inhalable LAMA/LABA combinations including dual monotherapies and fixed dose combinations; (3) report of acute exacerbations or mortality; (4) parallel, randomized controlled trial (RCT) study design, judged using the criteria of the Design Algorithm for Medical Literature on Intervention [29]; (5) treatment duration of 48 weeks or more; (6) human subjects; (7) publication in English.

### Primary and secondary outcomes

Our primary outcome was a comparison of the total exacerbation rate and all-cause mortality rate among LAMA/LABA combinations. Secondarily, we evaluated moderate to severe exacerbation rate, COPD-related mortality, cardiovascular disease-related mortality, major adverse cardiovascular events (MACE), and pneumonia.

### Information sources and search

We searched MEDLINE, EMBASE, and the Cochrane Central Register of Controlled Trials, following a pre-established study protocol and search strategy (search date: July 1, 2019). In addition, we referred to the US national library of medicine, the EU Clinical Trial Register, the AstraZeneca Clinical Trials website, the Boehringer Ingelheim clinical study results website, the GlaxoSmithKline Study Register, and the Novartis clinical trial results website. We also contacted authors and representatives of pharmaceutical companies, including GlaxoSmithKline, Boehringer Ingelheim, AstraZeneca, Novartis, and Kolon to obtain additional data. We conducted manual searches using the study identifiers or references of previous SRs. When designing this search strategy for SRs, we referred to the Peer Review of Electronic Search Strategies (PRESS) checklist [30]. The search terms were “COPD” AND inhaled drugs (“LAMA” AND “LABA”) AND randomized controlled design, which included controlled vocabulary and free text. The LAMAs included aclidinium, glycopyrrolate, tiotropium with a dry powder inhaler or soft mist inhaler, and umeclidinium. The LABAs included formoterol, indacaterol, olodaterol, salmeterol, and vilanterol. A detailed version of the search strategy can be found in both the Additional file 1 and PROSPERO.

### Study selection

We screened and reviewed studies according to the PRISMA flow diagram [31]. Duplicated studies were removed based on the title, abstract, and name of the authors. Independent reviewers (H.W.L./J.M.P.) conducted calibration exercises by title and abstract to improve inter-observer reliability, with a sample of 200 randomly selected studies (agreement = 97%, Cohen’s kappa = 0.81). The two reviewers individually screened the abstracts and titles of all potentially eligible studies and performed a full-text review to assess whether the screened studies met the pre-established eligibility criteria. Any conflicts or disagreements regarding study selection were resolved by referring to the original articles and discussing them with a third reviewer (C.H.L.).

### Data collection and data items

We coordinated the data collection methods and pre-piloted formats to assess study quality and synthesize the study outcomes. Independent reviewers (H.W.L./J.M.P.) extracted the following data items: (1) basic study information (e.g., year of study, study duration, device used for treatment, study outcomes, and number of patients included in intention-to-treat analysis); (2) baseline characteristics of the study population (e.g., age, sex, body mass index (BMI), smoking status, and ethnicity); (3) clinical information of the study population (e.g., time since COPD diagnosis, severity of COPD, mean post-bronchodilator forced expiratory volume in the first second (FEV1), history of total exacerbations in the past year, patients with a history of ≥ 2 total exacerbations or ≥ 1 severe exacerbations in the past year, modified medical research council dyspnea scale score, and COPD assessment test score); (4) study outcomes (e.g., number of patients experiencing any COPD exacerbation or number experiencing moderate to severe exacerbation, number of all-cause mortalities and cause of death, number of patients with MACEs, and number of patients with pneumonia until the last follow-up). If the absolute number of patients was not available, we recovered the raw data by digitization from the Kaplan–Meier curve of the time to first acute exacerbation [32]. The severity of COPD exacerbation was either assessed using the Exacerbations of Chronic Pulmonary Disease Tool [33] or estimated in terms of healthcare resource use [34]. Any controversy regarding the data extraction process was resolved by discussion.

### Network geometry

Two different network geometries were used in the present NMA. In network (A), the network meta-analysis was conducted under the assumption that there is a significant difference in efficacy and safety between individual drugs or their combinations within the same drug class. Network (A) expressed individual drugs or their combinations as nodes, and a direct comparison of two different treatments in an RCT as a link between nodes. In network (B), the network meta-analysis was conducted under the assumption that there was no difference in efficacy and safety between individual drugs or their combinations within the same drug class other than LAMA/LABA. Network (B) combined all inhaled treatments other than LAMA/LABA to each drug class (ICS/LAMA/LABA, ICS/LABA, LAMA, and LABA) and expressed them as each node. Network (B) was applied in the NMA of total exacerbation and all-cause mortality, which were the major outcomes of the present study. The number of direct treatment comparisons was expressed as the thickness of the edges between the nodes.

### Risk of bias within and across individual studies

Two reviewers (H.W.L./J.M.P.) independently appraised the risk of bias in each of the included studies in terms of the seven domains defined in the Cochrane Risk-of-Bias tool [35]. Any controversy regarding this risk of bias assessment was discussed with the other author (C.H.L.).

### Data synthesis and analysis

The present Bayesian NMA was conducted using a random effects model with a heterogeneous variance structure [36, 37] because we found more than two LAMA/LABA therapy regimens and assumed that the variance of the odds ratios of individual treatment (LAMA/LABA) compared with baseline treatment (Tiotropium) was different. The prior distributions of the Bayesian model parameters were assumed to be non-informative and to have normal or uniform distribution [38]. We estimated the relative probability of the best treatment based on the surface under the cumulative ranking curve (SUCRA) [38]. The median value of the posterior odds ratio (OR), with 95% credible intervals (CrIs), and the posterior probability of the OR exceeding 1 (*P*[OR > 1]) were estimated to identify the relationship between each inhaled drug and clinical outcomes. Statistical significance was defined when *P*(OR > 1) was less than 0.025 or more than 0.975. An OR greater than 1 in a pairwise comparison indicated that the comparator group (upper side of the league table) was more beneficial than the treatment group (left side of the league table). Additionally, pairwise meta-analyses were conducted using the random effects model for each direct comparison, and the results were presented as ORs with 95% confidence intervals (CIs). Sensitivity analysis was conducted according to network geometry (A and B) and cause of death (COPD-related and cardiovascular disease-related mortality).

The parameters were estimated using the Markov Chain Monte Carlo (MCMC) algorithm in WinBUGS version 1.4.6 (Imperial College and Medical Research Council, UK). Convergence of the MCMC algorithm was checked using trace plots, autocorrelation plots, and Gelman–Rubin statistics. We discarded the first 20,000 iterations to eliminate the initial value effect and selected 10,000 samples from the MCMC algorithm in two chains after applying the appropriate thinning rate to satisfy the autocorrelation assumption.

We reviewed the baseline characteristics of the eligible trials and the demographic characteristics of patients to monitor the homogeneity and similarity assumptions. Publication bias was investigated in the direct comparisons, which included ≥ 3 RCTs, using funnel plots and Egger’s test. The consistency assumption stating that the direct estimates might be consistent with indirect estimates is another main assumption in a NMA, and this was assessed using the node-splitting method [39]. Heterogeneity was assessed based on the posterior median of the standard deviation (SD) between the studies. An SD close to 0 indicates small heterogeneity, while an SD > 1 indicates substantial heterogeneity [40, 41].

### Certainty of evidence

The certainty of evidence was rated using the GRADE (Grading of Recommendations, Assessment, Development and Evaluations) approach [42].

### Role of the funding source

The funding source had no role in study design, data collection and analysis, decision to publish, or preparation of the manuscript.

## Results

### Study selection and network geometry

**Fig. 1:**
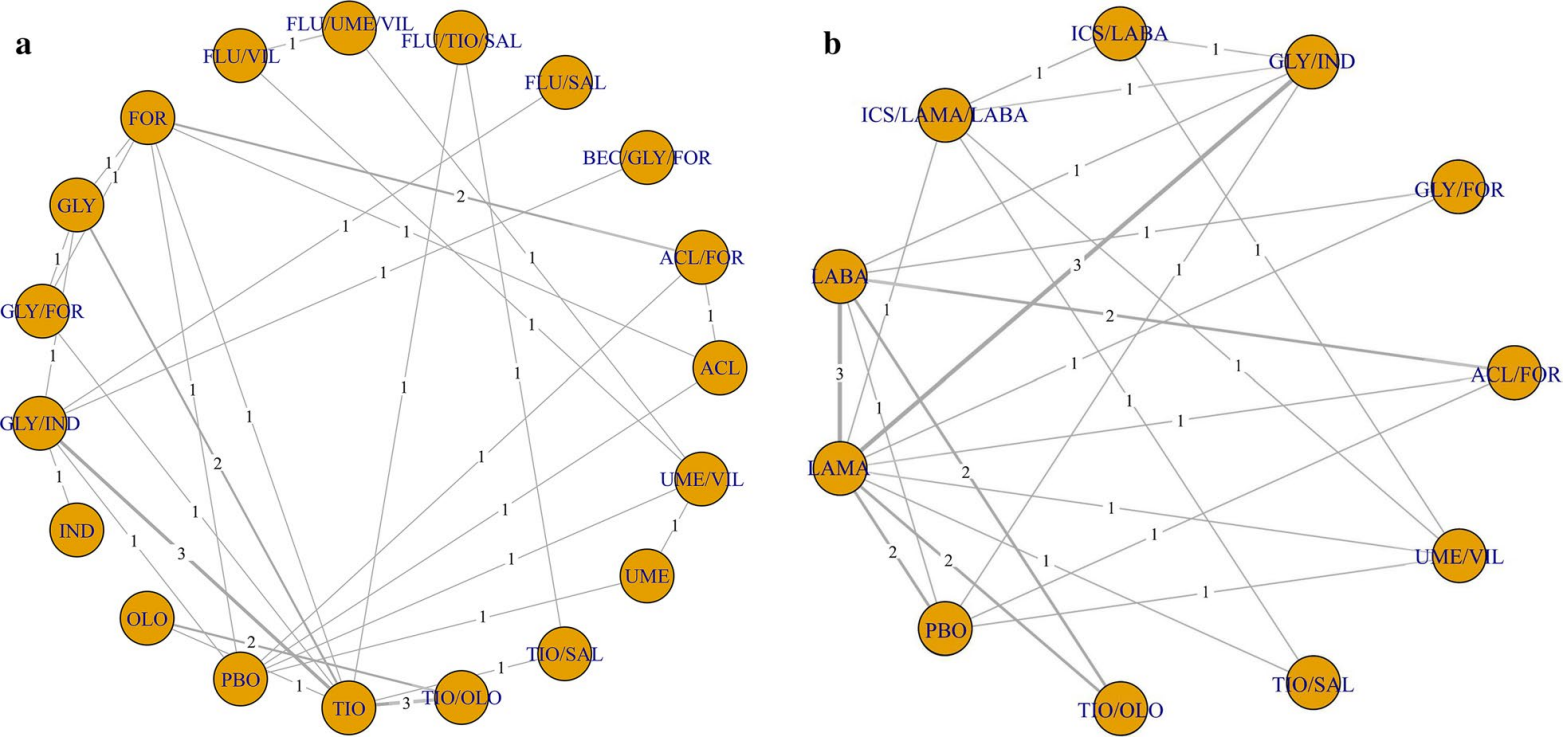
Network geometry of the direct comparison in the eligible 16 RCTs. Network (**a**) expressed individual drugs or their combinations as each node, and a direct comparison of two different treatments in an RCT was shown as a line between nodes. Network (**b**) combined all inhaled treatments other than individual LAMA/LABAs to each drug class (ICS/LAMA/LABA, ICS/LABA, LAMA, and LABA) and expressed them as each node. The number of direct comparison was expressed as a number in the middle of a line between nodes. *ACL* aclidinium, *BEC* beclomethasone, *FOR* formoterol, *FLU* fluticasone, *GLY* glycopyrrolate, *IND* indacaterol, *OLO* olodaterol, *PBO*, placebo, *SAL* salmeterol, *TIO* tiotropium, *UME* umeclidinium, *VIL* vilanterol

Among the total 5718 articles retrieved, 3696 were identified after the removal of duplicates, and 127 were found to be potentially relevant after screening by title and abstract (Additional file 2). After a full-text review, we found 16 articles that met the eligibility criteria of the present SR. The excluded articles are listed in the Additional file 3. Network geometries (A) and (B), addressing total exacerbations and all-cause mortality, respectively, are graphically expressed in Fig. 1.

### Study characteristics

The baseline characteristics of the eligible studies are summarized in Table 1. Among the 39,065 patients included in 16 RCTs published between 2007 and 2018, six LAMA/LABA combinations were identified; 6079 patients were in the tiotropium/olodaterol arm, 4334 were in glycopyrrolate/indacaterol arm, 2296 were in umeclidinium/vilanterol arm, 1060 were in aclidinium/formoterol arm, 1,035 were in glycopyrrolate/formoterol arm, and 148 were in tiotropium/salmeterol arm. The patients’ mean age was 64.8; 68.5% of them were men and 39.3% were current smokers. The dominant ethnicity was white or Caucasian. The patients’ mean post-bronchodilator FEV1 percentage was 45.6%, and no patients with mild COPD were found; specifically, eight RCTs enrolled patients with GOLD grade II–III, seven enrolled patients with GOLD grade II–IV, and two enrolled patients with GOLD grade III–IV. The treatment duration was 52 weeks in 15 RCTs and 64 weeks in one RCT. The patients used a dry powder inhaler in 12 RCTs, a soft mist inhaler in four RCTs, and a metered-dose inhaler in one RCT.

**Table 1:** Baseline characteristics of the included 16 studies

Author	Published year	Study ID	Number of patients	Age	Male (%)	Current smoker (%)	Ethnicity (%)	Mean post-BDR FEV1, %	GOLD stage	Previous exacerbation	Symptom	Intervention (LAMA/LABA)	Control	Treatment duration	Inhalation device
Aaron et al.	2007	ISRCTN29870041	449	67.7	56.4	27.9	White: 98.2%	41.8	II–IV	≥ 1	–	Tiotropium Handihaler/Salmeterol	1) Fluticasone/Tiotropium Handihaler/Salmeterol 2) Tiotropium Handihaler	52 weeks	DPI
Asai et al.	2013	NCT01285492	158	69.3	95.6	–	–	–	II–III	–	–	Glycopyrrolate/Indacaterol	Tiotropium Handihaler	52 weeks	DPI
Dahl et al.	2013	NCT01120717	338	62.6	76.9	45.2	Caucasian: 80.5% Asian: 19.5%	57.4	II–III	–	–	Glycopyrrolate/Indacaterol	Placebo	52 weeks	DPI
Wedzicha et al.	2013	NCT01120691	2206	63.3	74.8	37.6	White: 82.0% Asian: 11.7%	37.2	III–IV	≥ 1	–	Glycopyrrolate/Indacaterol	1) Glycopyrrolate 2) Tiotropium Handihaler	64 weeks	DPI
Donohue et al.	2014	NCT01316887	562	61.3	66.6	–	Non-hispanic: 92.8%	54.7	II–III	–	–	Umeclidinium/Vilanterol	1) Placebo 2) Umeclidinium	52 weeks	DPI
Buhl et al.	2015	NCT01431274 NCT01431287	5162	64	72.9	37	–	50	II–IV	–	–	Tiotropium Respimat/Olodaterol	1) Olodaterol 2) Tiotropium Respimat	52 weeks	SMI
Larbig et al.	2015	NCT01610037	812	64.4	72.4	–	–	–	II–III	–	–	Glycopyrrolate/Indacaterol	1) Placebo 2) Tiotropium Handihaler	52 weeks	DPI
Donohue et al.	2016	NCT01437540	590	64.2	55.1	45.9	White: 92.4%	51.4	II–III	–	–	Aclidinium/Formoterol	Formoterol	52 weeks	DPI
Ferguson et al.	2016	NCT01682863	615	63.6	65.6	50.9	White: 97.7%	54.4	II–III	–	mMRC ≥ 2	Glycopyrrolate/Indacaterol	Indacaterol	52 weeks	DPI
Wedzicha et al.	2016	NCT01782326	3358	64.5	76	39.7	–	44.1	II–III	≥ 1	mMRC ≥ 2	Glycopyrrolate/Indacaterol	Fluticasone/Salmeterol	52 weeks	DPI
D'Urzo et al.^b^	2017	NCT01572792	1669	64	53.1	51.5	White: 93.2%	53.5	II–III	–	–	Aclidinium/Formoterol	1) Formoterol 2) Placebo	52 weeks	DPI
Hanania et al.	2017	NCT01970878	3257	62.8	55.8	53.8	–	43.0^a^	II–IV	–	–	Glycopyrrolate/Formoterol	1) Formoterol 2) Glycopyrrolate 3) Tiotropium Handihaler	52 weeks	MDI
Ichinose et al.	2017	NCT01536262	122	69.8	95.9	27.9	–	57.5	II–IV	–	–	Tiotropium Respimat/Olodaterol	Olodaterol	52 weeks	SMI
Calverley et al.	2018	NCT02296138	7880	66.4	71.5	37	White: 79.5% Asian: 14.5%	44.5	II–IV	≥ 1	–	Tiotropium Respimat/Olodaterol	Tiotropium Respimat	52 weeks	SMI
Lipson et al.	2018	NCT02164513	10,355	65.3	66.4	34.6	White: 78.2% Asian: 16.0%	45.6	II–IV	≥ 1	CAT ≥ 10	Umeclidinium/Vilanterol	1) Fluticasone/Umeclidinium/Vilanterol 2) Fluticasone/Vilanterol	52 weeks	DPI
Papi et al.	2018	NCT02579850	1532	64.5	72	44.5	–	36.4	III–IV	≥ 1	CAT ≥ 10	Glycopyrrolate/Indacaterol	Beclometasone/Glycopyrrolate/Formoterol	52 weeks	DPI

*ID* identifier, *BDR* bronchodilator, *CAT* COPD assessment test, modified Medical Research Council: mMRC, *FEV1* forced expiratory volume in 1 s, *GOLD* Global Initiative for Chronic Obstructive Lung Disease, *LAMA* long-acting muscarinic antagonist, *LABA* long-acting beta-agonist, *DPI* dry powder inhaler, *SMI* soft mist inhaler, *MDI* metered dose inhaler

^a^As postBDR FEV1 data was not available, we described preBDR FEV1% value

^b^AUGMENT extension study maintained medications for additional 28 weeks after AUGMENT study was finished. The combined data from AUGMENT study (NCT01437397) and AUGMENT extension study (NCT01572792) were extracted

### Risk of bias within studies and across studies

The risk of bias was assessed and considered acceptable for our NMA (Additional file 4). No substantial risk of bias was detected in the random sequence generation or allocation concealment applied in the included studies. Blinding of participants and personnel was well conducted in most of the included RCTs. Our primary and secondary outcomes were unlikely to be influenced by incomplete outcome data because the reasons for withdrawal or follow-up loss were balanced and because outcome assessment was conducted with intention-to-treatment groups. Bias was rarely found from selective reporting of outcomes or any other sources. In analyses exploring the potential for risk of bias across studies, publication bias and selective reporting were not found (Additional file 5).

### Total exacerbations

**Fig. 2:**
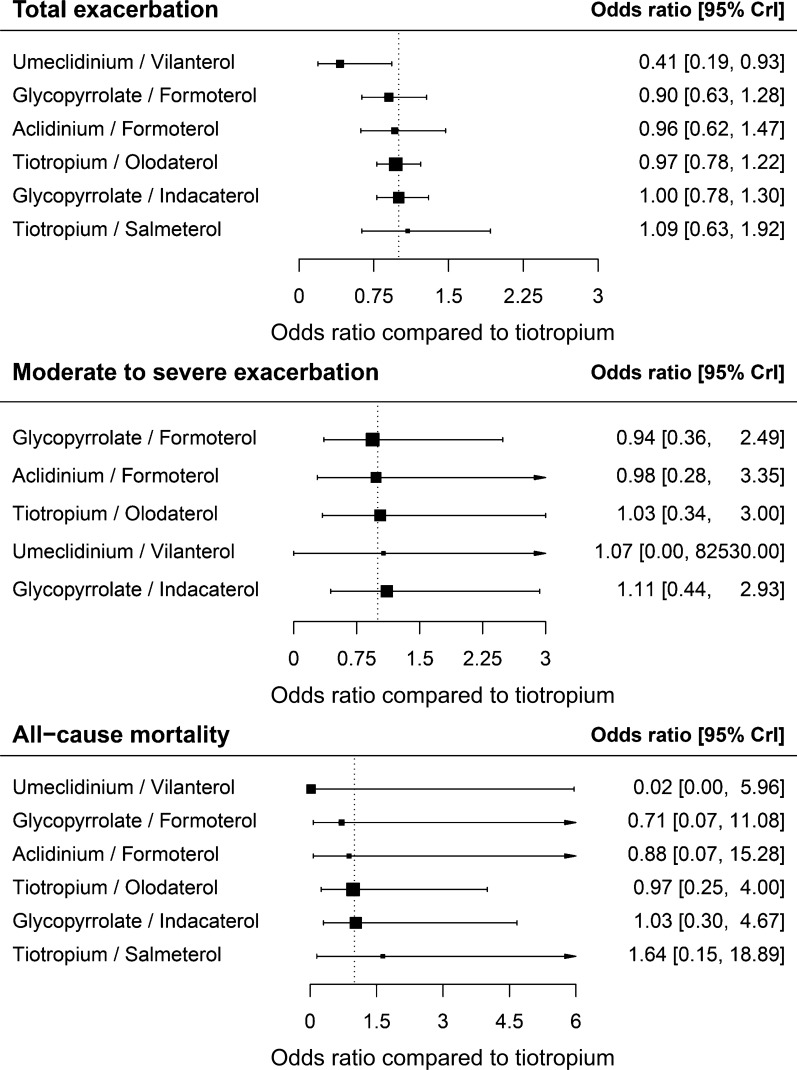
Forest plots of the risk of total exacerbations, moderate to severe exacerbations, and all-cause mortality. The risk of total exacerbations, moderate to severe exacerbations, and all-cause mortality were expressed as forest plots using the estimated odds ratios with 95% credible intervals compared to tiotropium. *CrI* credible interval

We analyzed 39,065 patients in 16 RCTs to compare efficacy among individual LAMA/LABA combinations in terms of reduction in total exacerbation. In this regard, umeclidinium/vilanterol was ranked first according to SUCRA, followed by glycopyrrolate/formoterol. Compared with tiotropium monotherapy, umeclidinium/vilanterol led to fewer exacerbations (Fig. 2). In addition, umeclidinium/vilanterol was significantly superior to tiotropium/olodaterol, aclidinium/formoterol, and glycopyrrolate/indacaterol with a moderate level of evidence, and tiotropium/salmeterol with a low level of evidence in terms of total exacerbation risk in network (A) (Table 2, Additional file 6). In network (B), umeclidinium/vilanterol no longer showed significant benefits in this regard (S7 information).

**Table 2:** Results of Bayesian network meta-analyses for exacerbation, mortality, and adverse events among LAMA and LABA combinations compared to tiotropium monotherapy in the network (A)

	Tiotropium/Olodaterol	Aclidinium/Formoterol	Umeclidinium/Vilanterol	Glycopyrrolate/Formoterol	Glycopyrrolate/Indacaterol	Tiotropium/Salmeterol
Total exacerbation (16 studies, 39,065 patients)						
Rank	3	4	1	2	5	6
SUCRA	0.438	0.4346	0.8802	0.5393	0.389	0.3016
NMA estimate OR (95% CrI)						
Tiotropium/Olodaterol	1					
Aclidinium/Formoterol	0.99 (0.6–1.6)	1				
Umeclidinium/Vilanterol	0.42 (0.19–0.97)*	0.43 (0.2–0.94)*	1			
Glycopyrrolate/Formoterol	0.92 (0.61–1.41)	0.94 (0.59–1.5)	2.18 (0.95–4.93)	1		
Glycopyrrolate/Indacaterol	1.02 (0.74–1.45)	1.04 (0.68–1.66)	2.43 (1.1–5.36)*	1.11 (0.76–1.66)	1	
Tiotropium/Salmeterol	1.12 (0.62–2.06)	1.14 (0.56–2.34)	2.66 (1–7.09)*	1.22 (0.62–2.37)	1.09 (0.59–2.02)	1
Moderate-to-severe exacerbation (7 studies, 27,489 patients)						
Rank	3	2	4	1	5	–
SUCRA	0.4689	0.5119	0.4639	0.5844	0.3794	–
NMA estimate OR (95% CrI)						
Tiotropium/Olodaterol	1					
Aclidinium/Formoterol	0.95 (0.18–5.04)	1				
Umeclidinium/Vilanterol	1.05 (0–91,490)	1.09 (0–97,430)	1			
Glycopyrrolate/Formoterol	0.9 (0.22–4.16)	0.95 (0.26–3.69)	0.86 (0–68,860)	1		
Glycopyrrolate/Indacaterol	1.08 (0.26–4.86)	1.13 (0.25–5.38)	1.04 (0–80,330)	1.19 (0.35–4.09)	1	
Tiotropium/Salmeterol	–	–	–	–	–	–
All-cause mortality (16 studies, 39,065 patients)						
Rank	4	3	1	2	5	6
SUCRA	0.4301	0.4567	0.8358	0.5097	0.4057	0.3083
NMA estimate OR (95% CrI)						
Tiotropium/Olodaterol	1					
Aclidinium/Formoterol	0.9 (0.05–21.92)	1				
Umeclidinium/Vilanterol	0.03 (0–7.15)	0.03 (0–6.88)	1			
Glycopyrrolate/Formoterol	0.74 (0.05–14.51)	0.82 (0.03–19.07)	29.98 (0.09–41,250)	1		
Glycopyrrolate/Indacaterol	1.05 (0.17–8.7)	1.19 (0.07–18.77)	42.73 (0.18–46,040)	1.43 (0.09–21.95)	1	
Tiotropium/Salmeterol	1.68 (0.1–27.64)	1.87 (0.04–59.48)	68.98 (0.16–92,860)	2.3 (0.06–59.11)	1.57 (0.08–22.96)	1
COPD-related mortality (7 studies, 25,900 patients)						
Rank	3	1	4	2	5	–
SUCRA	0.4741	0.7123	0.4046	0.6009	0.2731	–
NMA estimate OR (95% CrI)						
Tiotropium/Olodaterol	1					
Aclidinium/Formoterol	0.01 (0–1439)	1				
Umeclidinium/Vilanterol	1.93 (0–237,100)	274 (0–7,690,000,000)	1			
Glycopyrrolate/Formoterol	0.05 (0–75,270)	6.77 (0–858,000,000)	0.02 (0–1,910,000)	1		
Glycopyrrolate/Indacaterol	41.3 (0–10,600,000)	6241 (0–386,000,000,000)	22.16 (0–451,000,000)	1015 (0–2,200,000,000,000)	1	
Tiotropium/Salmeterol	–	–	–	–	–	–
Cardiovascular disease-related mortality (9 studies, 28,131 patients)						
Rank	4	1	2	5	3	–
SUCRA	0.3452	0.6024	0.4396	0.3087	0.3966	–
NMA estimate OR (95% CrI)						
Tiotropium/Olodaterol	1					
Aclidinium/Formoterol	0.04 (0–34)	1				
Umeclidinium/Vilanterol	0.34 (0–11,300)	9.08 (0–2,050,000)	1			
Glycopyrrolate/Formoterol	1.43 (0.02–244)	33.7 (0.08–125,700)	4.6 (0–158,000)	1		
Glycopyrrolate/Indacaterol	1.19 (0–1,010,000)	31 (0–134,000,000)	3.7 (0–41,900,000)	0.77 (0–912,000)	1	
Tiotropium/Salmeterol	–	–	–	–	–	–
Major adverse cardiovascular event (9 studies, 20,051 patients)						
Rank	3	1	–	2	4	–
SUCRA	0.3162	0.6174	–	0.5209	0.1683	–
NMA estimate OR (95% CrI)						
Tiotropium/Olodaterol	1					
Aclidinium/Formoterol	0.24 (0.01–8.83)	1				
Umeclidinium/Vilanterol	–	–	–			
Glycopyrrolate/Formoterol	0.4 (0.01–11.56)	1.64 (0.08–40.72)	–	1		
Glycopyrrolate/Indacaterol	1.94 (0.09–88.14)	8.06 (0.35–443)	–	4.88 (0.24–190.6)	1	
Tiotropium/Salmeterol	–	–	–	–	–	–
Pneumonia (16 studies, 39,065 patients)						
Rank	3	2	1	5	4	6
SUCRA	0.5538	0.5906	0.6822	0.4295	0.4599	0.155
NMA estimate OR (95% CrI)						
Tiotropium/Olodaterol	1					
Aclidinium/Formoterol	0.85 (0.13–5.22)	1				
Umeclidinium/Vilanterol	0.65 (0.04–11.25)	0.75 (0.05–12.24)	1			
Glycopyrrolate/Formoterol	1.26 (0.32–5.38)	1.49 (0.25–9.62)	1.94 (0.11–33.85)	1		
Glycopyrrolate/Indacaterol	1.17 (0.46–4.38)	1.4 (0.27–9.69)	1.84 (0.12–32.73)	0.93 (0.29–4.04)	1	
Tiotropium/Salmeterol	24.85 (0.13–164,500)	30.82 (0.12–224,300)	42.81 (0.09–341,300)	19.79 (0.1–137,800)	20.54 (0.11–132,100)	1

Median odds ratio with 95% credible interval was calculated as a row to column ratio. If the OR is significantly lower than 1, the drug in the left row is more beneficial than the other drug in the upper column

*CrI* credible interval, *NMA* network meta-analysis, *OR* odds ratio

* Indicates that the posterior probability is either less than 0.025 or more than 0.975, which is considered statistically significant

### Moderate to severe exacerbations

We analyzed 27,489 patients in seven RCTs to compare efficacy among individual LAMA/LABA combinations in terms of reducing moderate to severe exacerbations. Tiotropium/salmeterol was not analyzed because limited data were available. No significant difference occurred in terms of the ability of each LAMA/LABA combination to reduce moderate to severe exacerbations (Table 2).

### All-cause mortality

We analyzed 39,065 patients in 16 RCTs to compare efficacy among individual LAMA/LABA combinations in terms of reducing all-cause mortality. No LAMA/LABA combination was found to be superior to any others in terms of reducing all-cause mortality (network [A], Table 2; network (B), Additional file 7). In the sensitivity analyses of COPD-related and cardiovascular disease-related mortality, no significant results were found.

### Adverse events

We analyzed 20,051 patients in nine RCTs to compare the risk of MACE among individual LAMA/LABA combinations. Umeclidinium/vilanterol and tiotropium/salmeterol were not analyzed because limited data were available. We found no significant differences in the risk of MACE among LAMA/LABA combinations (Table 2). In addition, 39,065 patients in 16 RCTs were evaluated to compare the risk of pneumonia among different LAMA/LABA combinations. There was no significant difference in the risk of pneumonia among LAMA/LABA combinations (Table 2).

### Consistency assumption

The posterior effect size estimated by comparison in the present NMA was consistent with the results of the direct comparison approach (Additional file 8). In the inconsistency evaluation, most of the results satisfied the consistency assumption.

## Discussion

Our SR compared the efficacy and safety of LAMA/LABA combinations using Bayesian NMA. Umeclidinium/vilanterol was the most effective treatment in terms of reducing total exacerbation events among the LAMA/LABAs with low or moderate level of evidence, except glycopyrrolate/formoterol under the assumption that pharmacologic actions are different between individual drugs or their combinations within the same drug class (network (A)). However, no significant differences were observed under the assumption that pharmacologic actions are not different within the same drug class other LAMA/LABA (network (B)). Until now, it has not been clarified whether there are any differences in pharmacologic effects between individual drugs or their combinations within the same drug class other than LAMA/LABA in the treatment of COPD patients [43]. Furthermore, all-cause mortality, moderate-to-severe exacerbation, and the rate of adverse events were not different among the LAMA/LABA on our NMA. Therefore, our NMA suggests that there is no strong evidence suggesting different benefits among LAMA/LABAs in reducing the risk of exacerbation.

In previous NMAs, umeclidinium/vilanterol and glycopyrrolate/indacaterol showed better efficacy than aclidinium/formoterol for improving lung function [24, 25], while olodaterol/tiotropium showed better efficacy than umeclidinium/vilanterol for reducing symptoms [25]. In another NMA, umeclidinium/vilanterol showed better lung function compared to tiotropium/olodaterol, aclidinium/formoterol and tiotropium/formoterol, although that study should be interpreted with caution because it was commercially sponsored and its methodology was not described [44]. Another NMA compared the exacerbation and mortality risk among various inhaled drugs, but the only actual comparison between LAMA/LABA regimens was between glycopyrrolate/indacaterol and tiotropium/salmeterol, which revealed insignificant results [43]. In a recently published NMA, glycopyrronium/formoterol, glycopyrronium/indacaterol, aclidinium/formoterol, and umeclidinium/vilanterol showed similar efficacy in reducing exacerbation during 24 weeks of treatment, but lung function was more improved in glycopyrronium/formoterol group. However, our NMA including only RCTs with treatment duration ≥ 48 weeks showed the risk of acute exacerbation was significantly reduced in umeclidinium/vilanterol compared to tiotropium/olodaterol, aclidinium/formoterol, glycopyrrolate/indacaterol, and tiotropium/salmeterol.

There are differences in the onset of action, duration of effect, and specificity at the receptor or effector among LABAs [45] and LAMAs [18]. A previous NMA reported that indacaterol was more effective than other LABAs at improving trough FEV1 and symptoms [46], and two NMAs showed differences in treatment outcomes between LAMAs [25, 47]. Considering these results, it seems likely that different LAMA/LABAs have different clinical efficacy. In addition, combined LAMAs and LABAs may have synergistic actions [48, 49] that differ according to the combination used.

In the present study, when individual inhaled drugs or combination therapies were compared (network A), total exacerbation was more reduced in patients who used umeclidinium/vilanterol than in those who used other LAMA/LABAs. This result is consistent with previous studies. In a short-term head-to-head RCT, umeclidinium/vilanterol showed a better efficacy than tiotropium/olodaterol in improving trough FEV1 at week 8 [20]. However, the superiority of umeclidinium/vilanterol in reducing total exacerbation among different LAMA/LABAs disappeared in the analysis comparing individual LAMA/LABAs with drug classes (network [B]). Network (B) allows more nodes to be included in NMAs, but it assumes that all other drug class have equal efficacy and safety. In fact, glycopyrrolate/indacaterol was more effective than ICS/LABA in the FLAME trial, and ICS/LABA was more effective than umeclidinium/vilanterol in the IMPACT trial. The ICS/LABA combinations were different in those two studies, so the NMA using network (A) in the present study did not use the data from those studies when comparing glycopyrrolate/indacaterol with umeclidinium/vilanterol. However, the NMA using network (B) used both studies. Considering that the geometry of network (B) may be more desirable than that of network (A) in terms of reduced imprecision, we should be cautious before declaring the superiority of umeclidinium/vilanterol based on the results of the present NMA.

The present SR with NMA had several strengths. Firstly, to our knowledge, this study was a novel attempt to estimate the comparative efficacy and safety of various LAMA/LABA combinations. No previous NMAs primarily evaluated acute exacerbation, mortality, and adverse events because these outcomes are rare and would yield low statistical power. We believe that exacerbation and mortality are more direct clinical outcomes in patients with stable COPD, although lung function decline and respiratory symptoms are also important clinical outcomes. The present study used Bayesian methods to perform an appropriate analysis of rare events and estimated the value of SUCRA as a numeric presentation of the overall ranking associated with each treatment. It would not be a coincidence that the same agent (umeclidinium/vilanterol) ranked first in SUCRA in terms of reduction in total exacerbation and all-cause mortality. This result may constitute good evidence for generating new hypotheses. Secondly, the present study used pooled data from RCTs with a study duration ≥ 48 weeks. Previous NMAs have mainly focused on lung function decline and respiratory symptoms in RCTs within 24 weeks [24, 25]. In a pairwise meta-analysis conducted by Calzetta et al., treatment duration was an important factor affecting the efficacy of inhaled treatment to reduce respiratory symptoms [12]. Considering that COPD patients require lifelong treatment, an effect size estimated from longer-term clinical outcomes would more reliable when considering which inhaled treatment to prescribe. In our pilot study, pooling the different study periods together showed significant statistical discrepancies between the estimated outcome by indirect comparison and the outcome of the direct comparison. In fact, international industry guidance states that a treatment duration of at least 1 year is needed to modify exacerbations [50]. Through rigorous statistical methods, we found that the estimated results were more reliable when analyzed in RCTs conducted for ≥ 48 weeks. Third, our NMA used the two networks (A and B) under different assumptions that were mutually complementary. Currently, it has not been clearly demonstrated whether there are any differences in pharmacologic effects between individual drugs or their combinations within the same drug class other than LAMA/LABA in the treatment of COPD patients [43]. Network A has the same individual inhaled drug (eg. tiotropium) that mediates indirect comparison between different LAMA/LABAs, so it is more advantageous in terms of comparability or intransitivity. However, in network B, imprecision can be reduced in the network meta-analysis for all-cause mortality, because more studies can be included. Therefore, our study was able to take advantage of the two assumptions and make a balanced conclusion from the results of the two network meta-analyses.

There are limitations in our study. First, our meta-analysis included RCTs for patients with different characteristics, which may have a potential bias. For example, IMPACT trial showed a better efficacy of ICS/LABA than LAMA/LABA, while FLAME trial reported a better efficacy of LAMA/LABA than ICS/LABA. There are concerns that the contradicting results may depend on whether excluding asthmatics or not [51], and IMPACT trial did not exclude the patients with asthma history, which could favour ICS-containing treatment (ICS/LABA) compared with bronchodilators only (LAMA/LABA). However, it seems doubtable that asthma patients were actually enrolled in IMPACT trial more than FLAME trial, given that only 18% of participants in IMPACT trial revealed a positive bronchodilator reversibility (bronchodilator response > 12%/200 mL), while 45% of those in FLAME trial showed a positive bronchodilator reversibility. In addition, in our study, 6 RCTs enrolled only patients with more than one previous exacerbation history, but other studies included those with heterogeneous exacerbation history. Since previous exacerbation history was reported as the most important risk factor of exacerbation in ECLIPSE study [52], the comparisons between studies with different exacerbation history may affect results. Second, inconsistent results in the analyses for total exacerbation and moderate to severe exacerbation implied a between-study heterogeneity in regard to defining and classifying acute exacerbations. It was difficult to identify whether the method of defining and classifying exacerbations was identical in each RCT. Third, mortality and safety outcomes were evaluated by including only studies conducted for more than 48 weeks, but the number of events was small, resulting in extremely wide CIs. Therefore, no definite conclusion can be drawn from the data obtained on mortality and adverse events.

## Conclusions

Our NMA including all available RCTs showed that there is no strong evidence suggesting different benefits among LAMA/LABAs patients with stable COPD who have been followed up for 48 weeks or more. Physicians may choose any LAMA/LABA according to the availability or preference of individual patients in the treatment of stable COPD.

## Supplementary information

**Additional file 1.** Search strategy for the systematic review and network meta-analysis.

**Additional file 2.** PRISMA flow chart of the study selection for the network meta-analysis.

**Additional file 3.** List of excluded references after full-text review.

**Additional file 4.** The Cochrane Collaboration’s tool for assessing risk of bias for included randomized controlled trials.

**Additional file 5.** Assessment of publication bias in the direct comparisons including 3 or more studies.

**Additional file 6.** Estimates of effects and quality ratings for comparison of LAMA/LABA from Bayesian network meta-analyses.

**Additional file 7.** Results of Bayesian network meta-analyses for total exacerbation and all-cause mortality among LAMA and LABA combinations in the network (B).

**Additional file 8.** Consistency evaluation between Bayesian network meta-analyses and direct meta-analysis.

## Data Availability

The data are available by accessing the published studies listed in Table 1.

## Abbreviations

BMI: Body mass index
COPD: Chronic obstructive pulmonary disease
CrI: Credible interval
FEV1: Forced expiratory volume in the first second
LABA: Long-acting beta-agonist
LAMA: Long-acting muscarinic antagonist
MACE: Major adverse cardiovascular events
MCMC: Markov Chain Monte Carlo
NMA: Network meta-analysis
OR: Odds ratio
PRESS: Peer Review of Electronic Search Strategies
RCT: Randomized controlled trial
SD: Standard deviation
SR: Systematic review
SUCRA: Surface under the cumulative ranking curve
