# Supplementary material for: Comparisons of exacerbations and mortality among LAMA/LABA combinations in stable chronic obstructive pulmonary disease: systematic review and Bayesian network meta-analysis
Source: Respir Res. 2020 Nov 25;21:310. doi: 10.1186/s12931-020-01540-8 (PMC7687787; doi:10.1186/s12931-020-01540-8)
Supplement: Supplementary file 1 — Additional file 1. Search strategy for the systematic review and network meta-analysis. [file 12931_2020_1540_MOESM1_ESM.docx]

**Additional file 1. Search strategy for the systematic review and network meta-analysis**

**PUBMED (2019.7.1)**

**#1. COPD patients**

"Lung Diseases, Obstructive"[Mesh:noexp] OR "Pulmonary Disease, Chronic Obstructive"[Mesh] OR "Pulmonary Emphysema"[Mesh] OR “Bronchitis, chronic”[Mesh] OR **“**Chronic obstructive pulmonary disease"[tiab] OR "Emphysema"[tiab] OR "Chronic bronchitis"[tiab] OR "Chronic obstructive lung disease"[tiab] OR "Obstructive lung disease"[tiab] OR "Obstructive pulmonary disease"[tiab] OR "Obstructive lung diseases"[tiab] OR "Obstructive pulmonary diseases"[tiab] OR "COPD"[tiab]

107739

**#2. LABA (General)**

"adrenergic beta-2 receptor agonists"[Mesh] OR (("long-acting"[tiab] or "long acting"[tiab] or "ultra-long acting"[tiab] or "ultra-long-acting"[tiab]) **AND** (β agonist*[tiab] OR β-agonist*[tiab] OR β2 agonist*[tiab] OR β2-agonist*[tiab] OR β-2 agonist*[tiab] OR β-2-agonist*[tiab] OR B2 agonist*[tiab] OR B2-agonist*[tiab] OR B-2 agonist*[tiab] OR B-2-agonist*[tiab] OR β(2) agonist*[tiab] OR β(2)-agonist*[tiab] OR β adrenergic agonist*[tiab] OR β-adrenergic agonist*[tiab] OR β2 adrenergic agonist*[tiab] OR β2-adrenergic agonist*[tiab] OR β-2 adrenergic agonist*[tiab] OR β-2-adrenergic agonist*[tiab] OR B2 adrenergic agonist*[tiab] OR B2-adrenergic agonist*[tiab] OR B-2 adrenergic agonist*[tiab] OR B-2-adrenergic agonist*[tiab] OR β(2) adrenergic agonist*[tiab] OR β(2)-adrenergic agonist*[tiab] OR β adrenoceptor agonist*[tiab] OR β-adrenoceptor agonist*[tiab] OR β2 adrenoceptor agonist*[tiab] OR β2-adrenoceptor agonist*[tiab] OR β-2 adrenoceptor agonist*[tiab] OR β-2-adrenoceptor agonist*[tiab] OR B2 adrenoceptor agonist*[tiab] OR B2-adrenoceptor agonist*[tiab] OR B-2 adrenoceptor agonist*[tiab] OR B-2-adrenoceptor agonist*[tiab] OR β(2) adrenoceptor agonist*[tiab] OR β(2)-adrenoceptor agonist*[tiab] OR beta agonist*[tiab] OR beta-agonist*[tiab] OR beta2 agonist*[tiab] OR beta2-agonist*[tiab] OR beta-2 agonist*[tiab] OR beta-2-agonist*[tiab] OR beta(2) agonist*[tiab] OR beta(2)-agonist*[tiab] OR beta adrenergic agonist*[tiab] OR beta-adrenergic agonist*[tiab] OR beta2 adrenergic agonist*[tiab] OR beta2-adrenergic agonist*[tiab] OR beta-2 adrenergic agonist*[tiab] OR beta-2-adrenergic agonist*[tiab] OR beta(2) adrenergic agonist*[tiab] OR beta(2)-adrenergic agonist*[tiab] OR beta adrenoceptor agonist*[tiab] OR beta-adrenoceptor agonist*[tiab] OR beta2 adrenoceptor agonist*[tiab] OR beta2-adrenoceptor agonist*[tiab] OR beta-2 adrenoceptor agonist*[tiab] OR beta-2-adrenoceptor agonist*[tiab] OR beta(2) adrenoceptor agonist*[tiab] OR beta(2)-adrenoceptor agonist*[tiab])) OR "LABA"[tiab] OR "LABAs"[tiab]

4030

**#3. LAMA (General)**

"Muscarinic Antagonists"[Mesh] OR "Cholinergic Antagonists"[Mesh] OR (("long-acting"[tiab] or "long acting"[tiab] or "ultra-long acting"[tiab] or "ultra-long-acting"[tiab]) **AND** (muscarinic antagonist*[tiab] OR muscarinic receptor antagonist*[tiab] OR antimuscarinic agent*[tiab] OR anti-muscarinic agent*[tiab] OR muscarinic blocker*[tiab] OR muscarinic receptor blocker*[tiab] OR Cholinergic antagonist*[tiab] OR Cholinergic receptor antagonist*[tiab] OR anticholinergic agent*[tiab] OR anti-cholinergic agent*[tiab] OR cholinergic blocker*[tiab] OR cholinergic receptor blocker*[tiab])) OR "LAMA"[tiab] OR "LAMAs"[tiab]

17109

**#4. LABA (Individual drugs)**

"Formoterol fumarate"[Mesh] OR "formoterol"[tiab] OR "formoterol-fumarate"[tiab] OR "arformoterol"[tiab] OR "Indacaterol"[Supplementary Concept] OR "Indacaterol"[tiab] OR "Indacaterol-maleate"[tiab] OR "Olodaterol"[Supplementary Concept] OR "Olodaterol"[tiab] OR "Salmeterol Xinafoate"[Mesh] OR "Salmeterol"[tiab] OR "Salmeterol-xinafoate"[tiab] OR "Vilanterol"[Supplementary Concept] OR "Vilanterol"[tiab] OR "Vilanterol-trifenatate"[tiab] OR "Atock"[tiab] OR "Atimos"[tiab] OR "Foradil"[tiab] OR "Foradile"[tiab] OR "Oxeze"[tiab] OR "Oxis"[tiab] OR "Perforomist"[tiab] OR "Onbrez"[tiab] OR "Arcapta"[tiab] OR "QAB 149"[tiab] OR "QAB-149"[tiab] OR "QAB149"[tiab] OR "Striverdi"[tiab] OR "Aeromax"[tiab] OR "Arial"[tiab] OR "Asmerole"[tiab] OR "Astmerole"[tiab] OR "Beglan"[tiab] OR "Betamican"[tiab] OR "Dilamax"[tiab] OR "Inaspir"[tiab] OR "Salmetedur"[tiab] OR "Serevent"[tiab] OR "Ultrabeta"[tiab] OR "Ellipta"[tiab]

5317

**#5. LAMA (Individual drugs)**

"Aclidinium bromide"[Supplementary Concept] OR "aclidinium"[tiab] OR "aclidinium-bromide"[tiab] OR "Glycopyrrolate"[Mesh] OR "Glycopyrrolate"[tiab] OR "Glycopyrronium"[tiab] OR "Glycopyrronium-bromide"[tiab] OR "Tiotropium Bromide"[Mesh] OR "Tiotropium"[tiab] OR "Tiotropium-bromide"[tiab] OR "GSK573719"[Supplementary Concept] OR "Umeclidinium"[tiab] OR "Umeclidinium-bromide"[tiab] OR "Tudorza"[tiab] OR "Eklira"[tiab] OR "Bretaris"[tiab] OR "Seebri"[tiab] OR "Enurev"[tiab] OR "Tovanor"[tiab] OR "Spriva"[tiab] OR "Incruse"[tiab] OR "Incruse Ellipta"[tiab] OR "GSK573719"[tiab] OR "GSK-573719"[tiab]

3273

**#6. LABA/LAMA (Mixed drugs)**

(formoterol [tiab] AND aclidinium [tiab]) OR (formoterol [tiab] AND (glycopyrrolate [tiab] OR glycopyrronium [tiab])) OR (indacaterol [tiab] AND (glycopyrrolate [tiab] OR glycopyrronium [tiab])) OR (vilanterol [tiab] AND umeclidinium [tiab]) OR (olodaterol [tiab] AND tiotropium [tiab]) OR (formoterol [tiab] AND tiotropium [tiab]) OR "Duaklir"[tiab] OR "Brimica"[tiab] OR "Bevespi"[tiab] OR "Duova"[tiab] OR "Tioform"[tiab] OR "QVA149"[Supplementary Concept] OR "QVA149"[tiab] OR "Ultibro"[tiab] OR "Tiotropium-olodaterol" [Supplementary Concept] OR "Stiolto"[tiab] OR "Spiolto"[tiab] OR "Vahelva"[tiab] OR "Anoro"[tiab]

609

**#7. RCT Human**

(randomized controlled trial [pt] OR controlled clinical trial [pt] OR randomized [tiab] OR placebo [tiab] OR drug therapy [sh] OR randomly [tiab] OR trial [tiab] OR groups [tiab]) NOT (animals [mh] NOT humans [mh])

3929382

**#8. English**

English[Language]

25185323

**#1 AND #7 AND #8 AND (((#2 OR #4) AND (#3 OR #5)) OR #6)**

1010

**EMBASE (2019.7.1)**

**#1. COPD patients**

'Chronic obstructive lung disease'/exp OR 'Chronic bronchitis'/exp OR 'Lung emphysema'/exp OR 'Chronic obstructive pulmonary disease':ab,ti OR 'Emphysema':ab,ti OR 'Chronic bronchitis':ab,ti OR 'Chronic obstructive lung disease':ab,ti OR 'Obstructive lung disease':ab,ti OR 'Obstructive pulmonary disease':ab,ti OR 'Obstructive lung diseases':ab,ti OR 'Obstructive pulmonary diseases':ab,ti OR 'COPD':ab,ti

193190

**#2. LABA (General)**

'beta 2 adrenergic receptor stimulating agent'/exp OR ((‘long-acting’:ab,ti OR ‘long acting’:ab,ti OR ‘ultra-long acting’:ab,ti OR ‘ultra-long-acting’:ab,ti) **AND** (‘β agonist*’:ab,ti OR ‘β-agonist*’:ab,ti OR ‘β2 agonist*’:ab,ti OR ‘β2-agonist*’:ab,ti OR ‘β-2 agonist*’:ab,ti OR ‘β-2-agonist*’:ab,ti OR ‘B2 agonist*’:ab,ti OR ‘B2-agonist*’:ab,ti OR ‘B-2 agonist*’:ab,ti OR ‘B-2-agonist*’:ab,ti OR ‘β(2) agonist*’:ab,ti OR ‘β(2)-agonist*’:ab,ti OR ‘β adrenergic agonist*’:ab,ti OR ‘β-adrenergic agonist*’:ab,ti OR ‘β2 adrenergic agonist*’:ab,ti OR ‘β2-adrenergic agonist*’:ab,ti OR ‘β-2 adrenergic agonist*’:ab,ti OR ‘β-2-adrenergic agonist*’:ab,ti OR ‘B2 adrenergic agonist*’:ab,ti OR ‘B2-adrenergic agonist*’:ab,ti OR ‘B-2 adrenergic agonist*’:ab,ti OR ‘B-2-adrenergic agonist*’:ab,ti OR ‘β(2) adrenergic agonist*’:ab,ti OR ‘β(2)-adrenergic agonist*’:ab,ti OR ‘β adrenoceptor agonist*’:ab,ti OR ‘β-adrenoceptor agonist*’:ab,ti OR ‘β2 adrenoceptor agonist*’:ab,ti OR ‘β2-adrenoceptor agonist*’:ab,ti OR ‘β-2 adrenoceptor agonist*’:ab,ti OR ‘β-2-adrenoceptor agonist*’:ab,ti OR ‘B2 adrenoceptor agonist*’:ab,ti OR ‘B2-adrenoceptor agonist*’:ab,ti OR ‘B-2 adrenoceptor agonist*’:ab,ti OR ‘B-2-adrenoceptor agonist*’:ab,ti OR ‘β(2) adrenoceptor agonist*’:ab,ti OR ‘β(2)-adrenoceptor agonist*’:ab,ti OR ‘beta agonist*’:ab,ti OR ‘beta-agonist*’:ab,ti OR ‘beta2 agonist*’:ab,ti OR ‘beta2-agonist*’:ab,ti OR ‘beta-2 agonist*’:ab,ti OR ‘beta-2-agonist*’:ab,ti OR ‘beta(2) agonist*’:ab,ti OR ‘beta(2)-agonist*’:ab,ti OR ‘beta adrenergic agonist*’:ab,ti OR ‘beta-adrenergic agonist*’:ab,ti OR ‘beta2 adrenergic agonist*’:ab,ti OR ‘beta2-adrenergic agonist*’:ab,ti OR ‘beta-2 adrenergic agonist*’:ab,ti OR ‘beta-2-adrenergic agonist*’:ab,ti OR ‘beta(2) adrenergic agonist*’:ab,ti OR ‘beta(2)-adrenergic agonist*’:ab,ti OR ‘beta adrenoceptor agonist*’:ab,ti OR ‘beta-adrenoceptor agonist*’:ab,ti OR ‘beta2 adrenoceptor agonist*’:ab,ti OR ‘beta2-adrenoceptor agonist*’:ab,ti OR ‘beta-2 adrenoceptor agonist*’:ab,ti OR ‘beta-2-adrenoceptor agonist*’:ab,ti OR ‘beta(2) adrenoceptor agonist*’:ab,ti OR ‘beta(2)-adrenoceptor agonist*’:ab,ti)) OR ‘LABA’:ab,ti OR ‘LABAs’:ab,ti

84747

**#3. LAMA (General)**

'cholinergic receptor blocking agent'/exp OR 'muscarinic receptor blocking agent'/exp OR ((‘long-acting’:ab,ti or ‘long acting’:ab,ti or ‘ultra-long acting’:ab,ti or ‘ultra-long-acting’:ab,ti) **AND** (‘muscarinic antagonist*’:ab,ti OR ‘muscarinic receptor antagonist*’:ab,ti OR ‘antimuscarinic agent*’:ab,ti OR ‘anti-muscarinic agent*’:ab,ti OR ‘muscarinic blocker*’:ab,ti OR ‘muscarinic receptor blocker*’:ab,ti OR ‘cholinergic antagonist*’:ab,ti OR ‘cholinergic receptor antagonist*’:ab,ti OR ‘anticholinergic agent*’:ab,ti OR ‘anti-cholinergic agent*’:ab,ti OR ‘cholinergic blocker*’:ab,ti OR ‘cholinergic receptor blocker*’:ab,ti)) OR ‘LAMA’:ab,ti OR ‘LAMAs’:ab,ti

232806

**#4. LABA (Individual drugs)**

‘formoterol fumarate’/exp OR ‘formoterol’:ab,ti OR ‘formoterol-fumarate’:ab,ti OR ‘arformoterol’:ab,ti OR ‘indacaterol’/exp OR ‘indacaterol’:ab,ti OR ‘indacaterol-maleate’:ab,ti OR ‘olodaterol’/exp OR ‘olodaterol’:ab,ti OR ‘salmeterol xinafoate’/exp OR ‘salmeterol’:ab,ti OR ‘salmeterol-xinafoate’:ab,ti OR ‘vilanterol’/exp OR ‘vilanterol’:ab,ti OR ‘vilanterol-trifenatate’:ab,ti OR ‘Atock’:ab,ti OR ‘Atimos’:ab,ti OR ‘Foradil’:ab,ti OR ‘Foradile’:ab,ti OR ‘Oxeze’:ab,ti OR ‘Oxis’:ab,ti OR ‘Perforomist’:ab,ti OR ‘Onbrez’:ab,ti OR ‘Arcapta’:ab,ti OR ‘QAB 149’:ab,ti OR ‘QAB-149’:ab,ti OR ‘QAB149’:ab,ti OR ‘Striverdi’:ab,ti OR ‘Aeromax’:ab,ti OR ‘Arial’:ab,ti OR ‘Asmerole’:ab,ti OR ‘Astmerole’:ab,ti OR ‘Beglan’:ab,ti OR ‘Betamican’:ab,ti OR ‘Dilamax’:ab,ti OR ‘Inaspir’:ab,ti OR ‘Salmetedur’:ab,ti OR ‘Serevent’:ab,ti OR ‘Ultrabeta’:ab,ti OR ‘Ellipta’:ab,ti

9946

**#5. LAMA (Individual drugs)**

‘aclidinium bromide’/exp OR ‘aclidinium’:ab,ti OR ‘aclidinium-bromide’:ab,ti OR ‘glycopyrronium’/exp OR ‘glycopyrrolate’:ab,ti OR ‘glycopyrronium’:ab,ti OR ‘glycopyrronium-bromide’:ab,ti OR ‘tiotropium bromide’/exp OR ‘tiotropium’:ab,ti OR ‘tiotropium-bromide’:ab,ti OR ‘umeclidinium’/exp OR ‘umeclidinium’:ab,ti OR ‘umeclidinium-bromide’:ab,ti OR ‘Tudorza’:ab,ti OR ‘Eklira’:ab,ti OR ‘Bretaris’:ab,ti OR ‘Seebri’:ab,ti OR ‘Enurev’:ab,ti OR ‘Tovanor’:ab,ti OR ‘Spriva’:ab,ti OR ‘Incruse’:ab,ti OR ‘Incruse Ellipta’:ab,ti OR ‘GSK573719’:ab,ti OR ‘GSK-573719’:ab,ti

12669

**#6. LABA/LAMA (Mixed drugs)**

'aclidinium bromide plus formoterol fumarate'/exp OR (‘formoterol’:ab,ti AND ‘aclidinium’:ab,ti) OR 'formoterol fumarate plus glycopyrronium bromide'/exp OR (‘formoterol’:ab,ti AND (‘glycopyrrolate’:ab,ti OR ‘glycopyrronium’:ab,ti)) OR 'glycopyrronium bromide plus indacaterol'/exp OR (‘indacaterol’:ab,ti AND (‘glycopyrrolate’:ab,ti OR ‘glycopyrronium’:ab,ti)) OR 'umeclidinium plus vilanterol'/exp OR (‘vilanterol’:ab,ti AND ‘umeclidinium’:ab,ti) OR 'olodaterol plus tiotropium bromide'/exp OR (‘olodaterol’:ab,ti AND ‘tiotropium’:ab,ti) OR (‘formoterol’:ab,ti AND ‘tiotropium’:ab,ti) OR ‘Duaklir’:ab,ti OR ‘Brimica’:ab,ti OR ‘Bevespi’:ab,ti OR ‘Duova’:ab,ti OR ‘Tioform’:ab,ti OR ‘QVA149’:ab,ti OR ‘Ultibro’:ab,ti OR ‘Stiolto’:ab,ti OR ‘Spiolto’:ab,ti OR ‘Vahelva’:ab,ti OR ‘Anoro’:ab,ti

1671

**#7. RCT Human**

('crossover procedure':de OR 'double-blind procedure':de OR 'randomized controlled trial':de OR 'single-blind procedure':de OR (random* OR factorial* OR crossover* OR cross NEXT/1 over* OR placebo* OR doubl* NEAR/1 blind* OR singl* NEAR/1 blind* OR assign* OR allocat* OR volunteer*):de,ab,ti) NOT (animal/exp NOT human/exp)

2185930

**#8. English**

English:la

29653690

**#1 AND #7 AND #8 AND (((#2 OR #4) AND (#3 OR #5)) OR #6)**

3040

**COCHRANE (2019.7.1)**

**#1. COPD patients**

MeSH descriptor: [Pulmonary Disease, Chronic Obstructive] explode all trees

OR

Chronic obstructive pulmonary disease:ti,ab,kw OR Emphysema:ti,ab,kw OR Chronic bronchitis:ti,ab,kw OR Chronic obstructive lung disease:ti,ab,kw OR Obstructive lung disease:ti,ab,kw OR Obstructive pulmonary disease:ti,ab,kw OR Obstructive lung diseases:ti,ab,kw OR Obstructive pulmonary diseases:ti,ab,kw OR COPD:ti,ab,kw

22161

**#2. LABA (General)**

MeSH descriptor: [Adrenergic beta-2 Receptor Agonists] explode all trees

OR

((long-acting:ti,ab,kw OR long acting:ti,ab,kw OR ultra-long acting:ti,ab,kw OR ultra-long-acting:ti,ab,kw) AND ("β agonist*":ti,ab,kw OR "β-agonist*":ti,ab,kw OR "β2 agonist*":ti,ab,kw OR "β2-agonist*":ti,ab,kw OR "β-2 agonist*":ti,ab,kw OR "β-2-agonist*":ti,ab,kw OR "B2 agonist*":ti,ab,kw OR "B2-agonist*":ti,ab,kw OR "B-2 agonist*":ti,ab,kw OR "B-2-agonist*":ti,ab,kw OR "β(2) agonist*":ti,ab,kw OR "β(2)-agonist*":ti,ab,kw OR "β adrenergic agonist*":ti,ab,kw OR "β-adrenergic agonist*":ti,ab,kw OR "β2 adrenergic agonist*":ti,ab,kw OR "β2-adrenergic agonist*":ti,ab,kw OR "β-2 adrenergic agonist*":ti,ab,kw OR "β-2-adrenergic agonist*":ti,ab,kw OR "B2 adrenergic agonist*":ti,ab,kw OR "B2-adrenergic agonist*":ti,ab,kw OR "B-2 adrenergic agonist*":ti,ab,kw OR "B-2-adrenergic agonist*":ti,ab,kw OR "β(2) adrenergic agonist*":ti,ab,kw OR "β(2)-adrenergic agonist*":ti,ab,kw OR "β adrenoceptor agonist*":ti,ab,kw OR "β-adrenoceptor agonist*":ti,ab,kw OR "β2 adrenoceptor agonist*":ti,ab,kw OR "β2-adrenoceptor agonist*":ti,ab,kw OR "β-2 adrenoceptor agonist*":ti,ab,kw OR "β-2-adrenoceptor agonist*":ti,ab,kw OR "B2 adrenoceptor agonist*":ti,ab,kw OR "B2-adrenoceptor agonist*":ti,ab,kw OR "B-2 adrenoceptor agonist*":ti,ab,kw OR "B-2-adrenoceptor agonist*":ti,ab,kw OR "β(2) adrenoceptor agonist*":ti,ab,kw OR "β(2)-adrenoceptor agonist*":ti,ab,kw OR "beta agonist*":ti,ab,kw OR "beta-agonist*":ti,ab,kw OR "beta2 agonist*":ti,ab,kw OR "beta2-agonist*":ti,ab,kw OR "beta-2 agonist*":ti,ab,kw OR "beta-2-agonist*":ti,ab,kw OR "beta(2) agonist*":ti,ab,kw OR "beta(2)-agonist*":ti,ab,kw OR "beta adrenergic agonist*":ti,ab,kw OR "beta-adrenergic agonist*":ti,ab,kw OR "beta2 adrenergic agonist*":ti,ab,kw OR "beta2-adrenergic agonist*":ti,ab,kw OR "beta-2 adrenergic agonist*":ti,ab,kw OR "beta-2-adrenergic agonist*":ti,ab,kw OR "beta(2) adrenergic agonist*":ti,ab,kw OR "beta(2)-adrenergic agonist*":ti,ab,kw OR "beta adrenoceptor agonist*":ti,ab,kw OR "beta-adrenoceptor agonist*":ti,ab,kw OR "beta2 adrenoceptor agonist*":ti,ab,kw OR "beta2-adrenoceptor agonist*":ti,ab,kw OR "beta-2 adrenoceptor agonist*":ti,ab,kw OR "beta-2-adrenoceptor agonist*":ti,ab,kw OR "beta(2) adrenoceptor agonist*":ti,ab,kw OR "beta(2)-adrenoceptor agonist*":ti,ab,kw)) OR LABA:ti,ab,kw OR LABAs:ti,ab,kw

2397

**#3. LAMA (General)**

MeSH descriptor: [Muscarinic Antagonists] explode all trees

OR

MeSH descriptor: [Cholinergic Antagonists] explode all trees

OR

((“long-acting”:ti,ab,kw OR “long acting”:ti,ab,kw OR “ultra-long acting”:ti,ab,kw OR “ultra-long-acting”:ti,ab,kw) AND (“muscarinic antagonist*”:ti,ab,kw OR “muscarinic receptor antagonist*”:ti,ab,kw OR “antimuscarinic agent*”:ti,ab,kw OR “anti-muscarinic agent*”:ti,ab,kw OR “muscarinic blocker*”:ti,ab,kw OR “muscarinic receptor blocker*”:ti,ab,kw OR “Cholinergic antagonist*”:ti,ab,kw OR “Cholinergic receptor antagonist*”:ti,ab,kw OR “anticholinergic agent*”:ti,ab,kw OR “anti-cholinergic agent*”:ti,ab,kw OR “cholinergic blocker*”:ti,ab,kw OR “cholinergic receptor blocker*”:ti,ab,kw)) OR LAMA:ti,ab,kw OR LAMAs:ti,ab,kw

2123

**#4. LABA (Individual drugs)**

MeSH descriptor: [Formoterol Fumarate] explode all trees OR

MeSH descriptor: [Salmeterol Xinafoate] explode all trees

OR

"formoterol":ti,ab,kw OR "formoterol-fumarate":ti,ab,kw OR "arformoterol":ti,ab,kw OR "Indacaterol":ti,ab,kw OR "Indacaterol-maleate":ti,ab,kw OR "Olodaterol":ti,ab,kw OR "Salmeterol":ti,ab,kw OR "Salmeterol-xinafoate":ti,ab,kw OR "Vilanterol":ti,ab,kw OR "Vilanterol-trifenatate":ti,ab,kw OR "Atock":ti,ab,kw OR "Atimos":ti,ab,kw OR "Foradil":ti,ab,kw OR "Foradile":ti,ab,kw OR "Oxeze":ti,ab,kw OR "Oxis":ti,ab,kw OR "Perforomist":ti,ab,kw OR "Onbrez":ti,ab,kw OR "Arcapta":ti,ab,kw OR "QAB 149":ti,ab,kw OR "QAB-149":ti,ab,kw OR "QAB149":ti,ab,kw OR "Striverdi":ti,ab,kw OR "Aeromax":ti,ab,kw OR "Arial":ti,ab,kw OR "Asmerole":ti,ab,kw OR "Astmerole":ti,ab,kw OR "Beglan":ti,ab,kw OR "Betamican":ti,ab,kw OR "Dilamax":ti,ab,kw OR "Inaspir":ti,ab,kw OR "Salmetedur":ti,ab,kw OR "Serevent":ti,ab,kw OR "Ultrabeta":ti,ab,kw OR "Ellipta":ti,ab,kw

6891

**#5. LAMA (Individual drugs)**

MeSH descriptor: [Glycopyrrolate] explode all trees

OR

MeSH descriptor: [Tiotropium Bromide] explode all trees

OR

"aclidinium":ti,ab,kw OR "aclidinium-bromide":ti,ab,kw OR "Glycopyrrolate":ti,ab,kw OR "Glycopyrronium":ti,ab,kw OR "Glycopyrronium-bromide":ti,ab,kw OR "Tiotropium":ti,ab,kw OR "Tiotropium-bromide":ti,ab,kw OR "Umeclidinium":ti,ab,kw OR "Umeclidinium-bromide":ti,ab,kw OR "Tudorza":ti,ab,kw OR "Eklira":ti,ab,kw OR "Bretaris":ti,ab,kw OR "Seebri":ti,ab,kw OR "Enurev":ti,ab,kw OR "Tovanor":ti,ab,kw OR "Spriva":ti,ab,kw OR "Incruse":ti,ab,kw OR "Incruse Ellipta":ti,ab,kw OR "GSK573719":ti,ab,kw OR "GSK-573719":ti,ab,kw

3790

**#6. LABA/LAMA (Mixed drugs)**

(“formoterol”:ti,ab,kw AND “aclidinium”:ti,ab,kw) OR (“formoterol”:ti,ab,kw AND (“glycopyrrolate”:ti,ab,kw OR “glycopyrronium”:ti,ab,kw)) OR (“indacaterol”:ti,ab,kw AND (“glycopyrrolate”:ti,ab,kw OR “glycopyrronium”:ti,ab,kw)) OR (“vilanterol”:ti,ab,kw AND “umeclidinium”:ti,ab,kw) OR (“olodaterol”:ti,ab,kw AND “tiotropium”:ti,ab,kw) OR (“formoterol”:ti,ab,kw AND “tiotropium”:ti,ab,kw) OR "Duaklir":ti,ab,kw OR "Brimica":ti,ab,kw OR "Bevespi":ti,ab,kw OR "Duova":ti,ab,kw OR "Tioform":ti,ab,kw OR "QVA149":ti,ab,kw OR "Ultibro":ti,ab,kw OR "Stiolto":ti,ab,kw OR "Spiolto":ti,ab,kw OR "Vahelva":ti,ab,kw OR "Anoro":ti,ab,kw

1258

**#7. RCT Human**

Select “Trials”

**#1 AND #7 AND (((#2 OR #4) AND (#3 OR #5)) OR #6)**

1668
