# Supplementary figures and images for: Comparisons of exacerbations and mortality among LAMA/LABA combinations in stable chronic obstructive pulmonary disease: systematic review and Bayesian network meta-analysis
Source: Respir Res. 2020 Nov 25;21:310. doi: 10.1186/s12931-020-01540-8 (PMC7687787; doi:10.1186/s12931-020-01540-8)

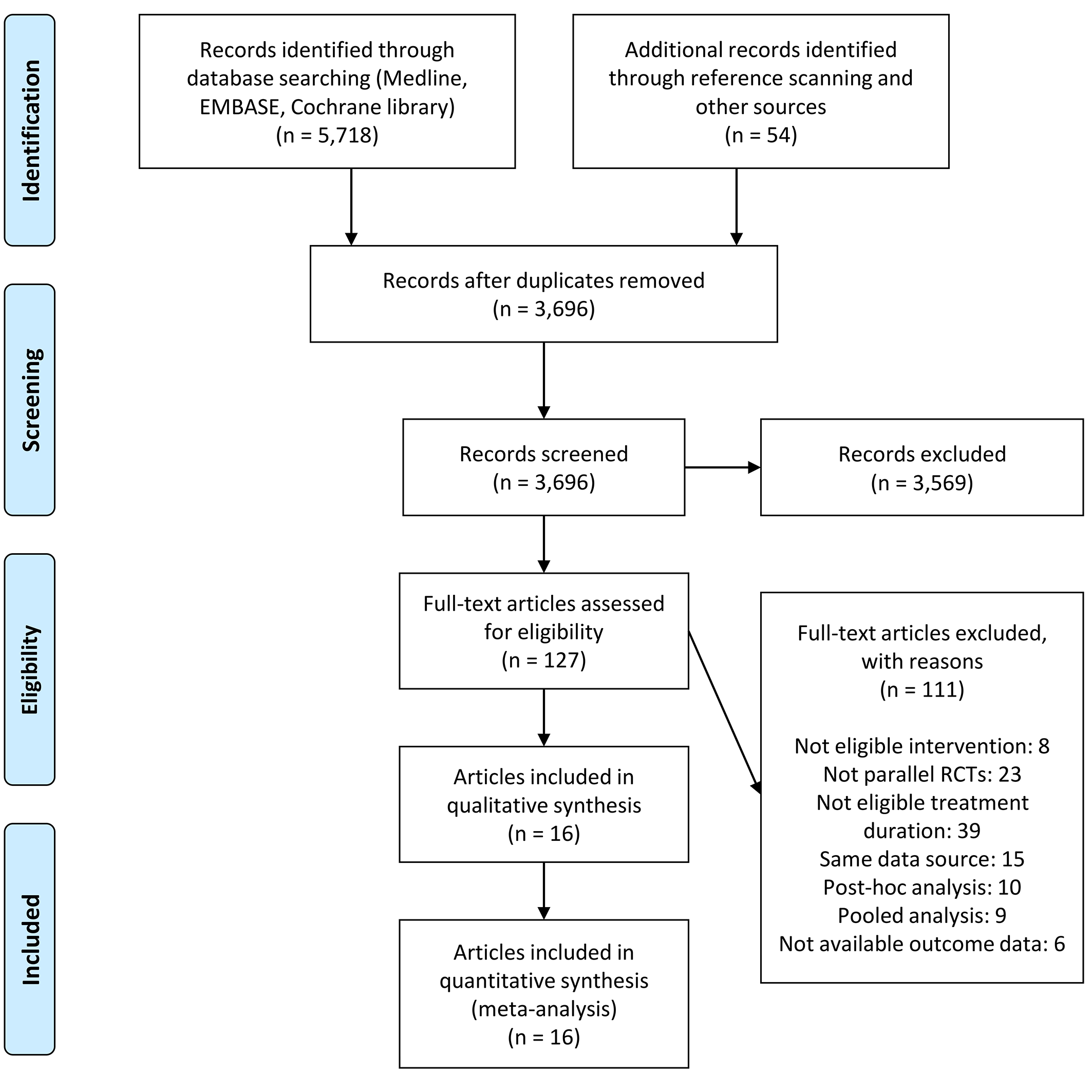

Supplement: Supplementary file 2 — Additional file 2. PRISMA flow chart of the study selection for the network meta-analysis. [file 12931_2020_1540_MOESM2_ESM.tif]
