# Supplementary material for: Comparisons of exacerbations and mortality among LAMA/LABA combinations in stable chronic obstructive pulmonary disease: systematic review and Bayesian network meta-analysis
Source: Respir Res. 2020 Nov 25;21:310. doi: 10.1186/s12931-020-01540-8 (PMC7687787; doi:10.1186/s12931-020-01540-8)
Supplement: Supplementary file 3 — Additional file 3. List of excluded references after full-text review. [file 12931_2020_1540_MOESM3_ESM.docx]

**Additional file 3. List of excluded references after full-text review**

| **Number** | **Title** | **First author** | **Journal (Year)** | **Main reason for exclusion** |
| --- | --- | --- | --- | --- |
| 1 | Randomized, Double-Blind, Dose-Finding Study for Tiotropium when Added to Olodaterol, Administered via the Respimat® Inhaler in Patients with Chronic Obstructive Pulmonary Disease | Aalber et al. | Advances in Therapy (2015) | This study was not designed as a parallel group RCT. |
| 2 | The efficacy and safety of umeclidinium/vilanterol compared with tiotropium or vilanterol over 24 weeks in subjects with COPD | Anzueto et al. | American Journal of Respiratory and Critical Care Medicine (2013) (Abstract) | This study was eligible to be included in our study, but we used another reference with the same data source. |
| 3 | The effect of indacaterol/glycopyrronium versus tiotropium or salmeterol/fluticasone on the prevention of clinically important deterioration in COPD | Anzueto et al. | International Journal of Chronic Obstructive Pulmonary Disease (2017) | This study was a pooled analysis of previous RCTs. |
| 4 | Indacaterol/glycopyrronium versus salmeterol/fluticasone in the prevention of clinically important deterioration in COPD: results from the FLAME study | Anzueto et al. | Respiratory Research (2018) | This study was a post-hoc analysis using previously published data. |
| 5 | Efficacy and safety of indacaterol/glycopyrronium in Japanese patients with COPD: pooled analysis of SHINE and ARISE | Asai et al. | Respiratory Investigation (2016) | This study was a pooled analysis of previous RCTs. |
| 6 | Use of the Daily PROactive instrument to evaluate physical activity in patients with COPD: results from ACTIVATE | Aymerich et al. | European Respiratory Journal (2017) (Abstract) | The intervention duration of this study was less than 48 weeks. |
| 7 | Lung function and long-term safety of tiotropium/olodaterol in East Asian patients with chronic obstructive pulmonary disease | Bai et al. | International Journal of Chronic Obstructive Pulmonary Disease (2017) | This study was a post-hoc analysis using previously published data. |
| 8 | Concurrent use of indacaterol plus tiotropium in patients with COPD provides superior bronchodilation compared with tiotropium alone: a randomised, double-blind comparison | Balkissoon et al. | Thorax (2012) | This study was eligible for our study, but we used another reference with the same data source. |
| 9 | Dual bronchodilation with QVA149 versus single bronchodilator therapy: the SHINE study | Bateman et al. | European Respiratory Journal (2013) | The intervention duration of this study was less than 48 weeks. |
| 10 | Withdrawal of inhaled corticosteroids and exacerbations of COPD: the wisdom phenotyping substudy | Bateman et al. | American Journal of Respiratory and Critical Care Medicine (2015) (Abstract) | Study intervention did not meet eligibility criteria of our study. |
| 11 | Aclidinium bromide and formoterol fumarate as a fixed-dose combination in COPD: pooled analysis of symptoms and exacerbations from two six-month, multicentre, randomised studies (ACLIFORM and AUGMENT) | Bateman et al. | Respiratory Research (2015) | This study was a pooled analysis of previous RCTs. |
| 12 | Effect of QVA149 on lung volumes and exercise tolerance in COPD patients: the BRIGHT study | Beeh et al. | Respiratory Medicine (2014) | This study was not designed as a parallel group RCT. |
| 13 | ENERGITO: Efficacy and safety of once-daily combined tiotropium + olodaterol versus twice-daily combined fluticasone propionate + salmeterol | Beeh et al. | European Respiratory Journal 2015 46: PA4366 (Abstract) | This study was not designed as a parallel group RCT. |
| 14 | The lung function profile of once-daily tiotropium and olodaterol via respimat® is superior to that of twice-daily salmeterol and fluticasone propionate via a accuhaler® (Energito® study) | Beeh et al. | International Journal of Chronic Obstructive Pulmonary Disease (2016) | This study was not designed as a parallel group RCT. |
| 15 | Efficacy and safety of once-daily QVA149 compared with the free combination of once-daily tiotropium plus twice-daily formoterol in patients with moderate-to-severe COPD (QUANTIFY): a randomised, non-inferiority study | Buhl et al. | Thorax (2015) | The intervention duration of this study was less than 48 weeks. |
| 16 | Long-term general and cardiovascular safety of tiotropium/olodaterol in patients with moderate to very severe chronic obstructive pulmonary disease | Buhl et al. | Respiratory Medicine (2017) | This study was eligible for our study, but we used another reference with the same data source. |
| 17 | A 24-week randomized, double-blind, placebo-controlled study of the efficacy and safety of once-daily umeclidinium/vilanterol 125/25 mcg in COPD | Celli et al. | American Journal of Respiratory and Critical Care Medicine (2013) (Abstract) | This study was eligible for our study, but we used another reference with the same data source. |
| 18 | Once-daily umeclidinium/vilanterol 125/25 μg therapy in COPD | Celli et al. | Chest (2014) | The intervention duration of this study was less than 48 weeks. |
| 19 | The addition of salmeterol 50 microg bid to anticholinergic treatment in patients with COPD: a randomized, placebo controlled trial. Chronic obstructive pulmonary disease | Chapman et al. | Canadian Respiratory Journal (2002) | The study intervention did not meet the eligibility criteria of our study. |
| 20 | QVA149 Improves Lung Function, Dyspnea, and Health Status Independent of Previously Prescribed Medications and COPD Severity: A Subgroup Analysis from the SHINE and ILLUMINATE Studies | Chapman et al. | COPD (2015) | This study was a post-hoc analysis using previously published data. |
| 21 | Long-term triple therapy de-escalation to indacaterol/ glycopyrronium in patients with chronic obstructive pulmonary disease (sunset): A randomized, double-blind, triple-dummy clinical trial | Chapman et al. | American Journal of Respiratory and Critical Care Medicine (2018) | The intervention duration of this study was less than 48 weeks. |
| 22 | Efficacy and safety of QVA149 compared to the concurrent administration of its monocomponents indacaterol and glycopyrronium: The BEACON study | Dahl et al. | International Journal of Chronic Obstructive Pulmonary Disease (2013) | The intervention duration of this study was less than 48 weeks. |
| 23 | A randomized study using functional respiratory imaging to characterize bronchodilator effects of glycopyrrolate/formoterol fumarate delivered by a metered dose inhaler using co-suspension delivery technology in patients with COPD | De Backer et al. | International Journal of Chronic Obstructive Pulmonary Disease (2018) | The intervention duration of this study was less than 48 weeks. |
| 24 | Efficacy and safety of umeclidinium plus vilanterol versus tiotropium, vilanterol, or umeclidinium monotherapies over 24 weeks in patients with chronic obstructive pulmonary disease: results from two multicentre, blinded, randomised controlled trials | Decramer et al. | Lancet Respiratory Medicine (2014) | The intervention duration of this study was less than 48 weeks. |
| 25 | Long-term safety and tolerability of umeclidinium/vilanterol and umeclidinium in COPD | Donohue et al. | European Respiratory Journal (2013) (Abstract) | This study was eligible for our study, but we used another reference with the same data source. |
| 26 | Efficacy and safety of once-daily umeclidinium/vilanterol 62.5/25 mcg in COPD | Donohue et al. | Respiratory Medicine (2013) | The intervention duration of this study was less than 48 weeks. |
| 27 | Improvements in lung function with umeclidinium/vilanterol versus fluticasone propionate/salmeterol in patients with moderate-to-severe COPD and infrequent exacerbations | Donohue et al. | Respiratory Medicine (2015) | The intervention duration of this study was less than 48 weeks. |
| 28 | Magnitude of umeclidinium/vilanterol lung function effect depends on monotherapy responses: results from two randomised controlled trials | Donohue et al. | Respiratory Medicine (2016) | This study was not designed as a parallel group RCT. |
| 29 | Clinical efficacy and safety of formoterol and tiotropium administration in patient with chronic heart failure due to coronary artery disease combined with chronic obstructive pulmonary disease | Evdokimov et al. | European Journal of Heart Failure (2015) (Abstract) | This reference did not describe the exacerbation rate or mortality rate, which were the outcomes of our meta-analysis. |
| 30 | Prolonged use (18 months) of tiotropium and indacaterol in patient with chronic heart failure due to coronary artery disease combined with chronic obstructive pulmonary disease | Evdokimov et al.2 | European Journal of Heart Failure (2016) (Abstract) | This reference did not describe the exacerbation rate or mortality rate, which were the outcomes of our meta-analysis. |
| 31 | Dose-response to inhaled glycopyrrolate delivered with a novel Co-Suspension™ Delivery Technology metered dose inhaler (MDI) in patients with moderate-to-severe COPD | Fabbri et al. | Respiratory Research (2016) | This study was eligible for our study, but we used another reference with the same data source. |
| 32 | Comparative Efficacy of Once-Daily Umeclidinium/Vilanterol and Tiotropium/Olodaterol Therapy in Symptomatic Chronic Obstructive Pulmonary Disease: a Randomized Study | Feldman et al. | Advances in Therapy (2017) | This study was not designed as a parallel group RCT. |
| 33 | Efficacy and safety of tiotropium + olodaterol maintenance treatment in patients with copd in the TONADO® and OTEMTO® studies: A subgroup analysis by age | Ferguson et al. | International Journal of Chronic Obstructive Pulmonary Disease (2016) | This study was a post-hoc analysis using previously published data. |
| 34 | Effect of tiotropium and olodaterol on symptoms and patient-reported outcomes in patients with COPD: Results from four randomised, double-blind studies | Ferguson et al. | NPJ Primary Care Respiratory Medicine (2017) | This study was a pooled analysis of previous RCTs. |
| 35 | Triple therapy with budesonide/glycopyrrolate/formoterol fumarate with co-suspension delivery technology versus dual therapies in chronic obstructive pulmonary disease (KRONOS): a double-blind, parallel-group, multicentre, phase 3 randomised controlled trial | Ferguson et al. | Lancet Respiratory Medicine (2018) | The intervention duration of this study was less than 48 weeks. |
| 36 | Pharmacokinetics, safety, and tolerability of aclidinium/formoterol fixed dose combination via pressair/genuair vs formoterol via foradil aerolizer in patients with moderate to severe COPD | Fogarty et al. | Chest (2014) (Abstract) | This reference did not describe the exacerbation rate or mortality rate, which were the outcomes of our meta-analysis. |
| 37 | Capturing Exacerbations of Chronic Obstructive Pulmonary Disease with EXACT. A Subanalysis of FLAME | Frent et al. | American Journal of Respiratory and Critical Care Medicine (2019) | This study was a post-hoc analysis using previously published data. |
| 38 | Benefits of dual bronchodilation with QVA149 once daily versus placebo, indacaterol, NVA237 and tiotropium in patients with COPD: the Shine study | Frith et al. | Respirology (2013) (Abstract) | This study was eligible for our study, but we used another reference with the same data source. |
| 39 | Glycopyrronium once-daily significantly improves lung function and health status when combined with salmeterol/fluticasone in patients with COPD: the GLISTEN study, a randomised controlled trial | Frith et al. | Thorax (2015) | The study intervention did not meet the eligibility criteria of our study. |
| 40 | Efficacy and safety of the direct switch to indacaterol/glycopyrronium from salmeterol/fluticasone in non-frequently exacerbating COPD patients: The FLASH randomized controlled trial | Frith et al. | Respirology (2018) | The intervention duration of this study was less than 48 weeks. |
| 41 | Efficacy of tiotropium and indacaterol monotherapy and their combination on dynamic lung hyperinflation in COPD: a random open-label crossover study | Fujimoto et al. | International Journal of Chronic Obstructive Pulmonary Disease (2017) | This study was not designed as a parallel group RCT. |
| 42 | Indacaterol/glycopyrronium reduces the risk of clinically important deterioration in patients with moderate COPD: results from the crystal study | Greulich et al. | European Respiratory Journal (2017) (Abstract) | The study intervention did not meet the eligibility criteria of our study. |
| 43 | Indacaterol/glycopyrronium reduces the risk of clinically important deterioration after direct switch from baseline therapies in patients with moderate COPD: a post hoc analysis of the CRYSTAL study | Greulich et al. | International Journal of Chronic Obstructive Pulmonary Disease (2018) | This study was a post-hoc analysis using previously published data |
| 44 | Long-term safety and efficacy of glycopyrrolate/formoterol metered dose inhaler using novel Co-Suspension™ Delivery Technology in patients with chronic obstructive pulmonary disease | Hanania et al. | Respiratory Medicine (2017) | This study was eligible for our study, but we used another reference with the same data source. |
| 45 | Efficacy and safety of indacaterol/glycopyrronium in Japanese patients with COPD: a subgroup analysis from the SHINE study | Hashimoto et al. | International Journal of Chronic Obstructive Pulmonary Disease (2016) | This study was a post-hoc analysis using previously published data. |
| 46 | Effect of lung deflation with indacaterol plus glycopyrronium on ventricular filling in patients with hyperinflation and COPD (CLAIM): a double-blind, randomised, crossover, placebo-controlled, single-centre trial | Hohlfeld et al. | Lancet Respiratory Medicine (2018) | This study was not designed as a parallel group RCT. |
| 47 | Comparison of airway dimensions with once daily tiotropium plus indacaterol versus twice daily Advair in chronic obstructive pulmonary disease | Hoshino et al. | Pulmonary Pharmacology and Therapeutics (2015) | This reference did not describe the exacerbation rate or mortality rate, which were the outcomes of our meta-analysis. |
| 48 | The efficacy and safety of combined tiotropium and olodaterol via the Respimat(®) inhaler in patients with COPD: results from the Japanese sub-population of the Tonado(®) studies | Ichinose et al. | International Journal of Chronic Obstructive Pulmonary Disease (2016) | This study was a post-hoc analysis using previously published data. |
| 49 | Study Design of VESUTO®: Efficacy of Tiotropium/Olodaterol on Lung Hyperinflation, Exercise Capacity, and Physical Activity in Japanese Patients with Chronic Obstructive Pulmonary Disease | Ichinose et al. | Advances in Therapy (2017) | This study was not designed as a parallel group RCT. |
| 50 | Efficacy of tiotropium/olodaterol on lung volume, exercise capacity, and physical activity | Ichinose et al. | International Journal of Chronic Obstructive Pulmonary Disease (2018) | This study was not designed as a parallel group RCT. |
| 51 | Combinations of long acting β2 agonists to tiotropium: A randomized, double-blind, placebo-controlled, active-drug controlled, parallel design academic clinical trial in moderate COPD male patients | Imran et al. | Archives of Pharmacy Practice (2015) | The intervention duration of this study was less than 48 weeks. |
| 52 | Combined therapy with tiotropium and formoterol in chronic obstructive pulmonary disease: effect on the 6-minute walk test | Jayaram et al. | COPD (2013) | This study was not designed as a parallel group RCT. |
| 53 | Dual Bronchodilator Therapy with Umeclidinium/Vilanterol Versus Tiotropium plus Indacaterol in Chronic Obstructive Pulmonary Disease: a Randomized Controlled Trial | Kalberg et al. | Drugs in R&D (2016) | The intervention duration of this study was less than 48 weeks. |
| 54 | The impact of indacaterol/glycopyrronium fixed-dose combination versus tiotropium monotherapy on lung function and treatment preference: A randomized crossover study – the FAVOR study | Kardos et al. | International Journal of Chronic Obstructive Pulmonary Disease (2017) | This study was not designed as a parallel group RCT. |
| 55 | Dose-ranging study of 2 fixed-dose combinations of twice-daily aclidinium bromide plus formoterol in patients with moderate to severe COPD | Kerwin et al. | Chest (2013) (Abstract) | This study was not designed as a parallel group RCT. |
| 56 | Umeclidinium/vilanterol as step-up therapy from tiotropium in patients with moderate COPD: a randomized, parallel-group, 12-week study | Kerwin et al. | International Journal of Chronic Obstructive Pulmonary Disease (2017) | The intervention duration of this study was less than 48 weeks. |
| 57 | Dual Bronchodilation with Indacaterol Maleate/Glycopyrronium Bromide Compared with Umeclidinium Bromide/Vilanterol in Patients with Moderate-to-Severe COPD: results from Two Randomized, Controlled, Cross-over Studies | Kerwin et al. | Lung (2017) | This study was eligible for our study, but we used another reference with the same data source. |
| 58 | Effect of twice-daily aclidinium/formoterol versus monotherapy or tiotropium on 24-hour bronchodilation and symptom control in patients with COPD: results from amplify | Kerwin et al. | American Journal of Respiratory and Critical Care Medicine (2018) (Abstract) | This study was eligible for our study, but we used another reference with the same data source. |
| 59 | Changes in lung function and health status in patients with COPD treated with tiotropium or salmeterol plus fluticasone | Kurashima et al. | Respirology (2009) | This study was not designed as a parallel group RCT. |
| 60 | A randomized trial to determine the impact of indacaterol/glycopyrronium on nighttime oxygenation and symptoms in patients with moderate-to-severe COPD: the DuoSleep study | Lehmann et al. | International Journal of Chronic Obstructive Pulmonary Disease (2019) | This study was not designed as a parallel group RCT. |
| 61 | Improved lung function and patient-reported outcomes with co-suspension delivery technology glycopyrrolate/formoterol fumarate metered dose inhaler in COPD: a randomized Phase III study conducted in Asia, Europe, and the USA | Lipworth et al. | International Journal of Chronic Obstructive Pulmonary Disease (2018) | The intervention duration of this study was less than 48 weeks. |
| 62 | Glycopyrrolate/Formoterol Fumarate Fixed-Dose Combination Delivered by Metered Dose Inhaler Improves Lung Function and Symptoms in Patients with COPD: Results from the PINNACLE-4 Study in Asia, Europe, and the USA | Lipworth et al. | American Journal of Respiratory and Critical Care Medicine (2018) (Abstract) | The study intervention did not meet the eligibility criteria of our study. |
| 63 | Withdrawal of inhaled glucocorticoids and exacerbations of COPD | Magnussen et al. | The New England Journal of Medicine (2014) | The intervention duration of this study was less than 48 weeks. |
| 64 | Concurrent use of indacaterol plus tiotropium in patients with COPD provides superior bronchodilation compared with tiotropium alone: A randomised, double-blind comparison | Mahler et al. | Thorax (2012) | This study was not designed as a parallel group RCT. |
| 65 | Dual bronchodilation with QVA149 reduces patient-reported dyspnoea in COPD: The BLAZE study | Mahler et al. | European Respiratory Journal (2014) | The intervention duration of this study was less than 48 weeks. |
| 66 | FLIGHT1 and FLIGHT2: Efficacy and safety of QVA149 (indacaterol/glycopyrrolate) versus its monocomponents and placebo in patients with chronic obstructive pulmonary disease | Mahler et al. | American Journal of Respiratory and Critical Care Medicine (2015) | This study was a post-hoc analysis using previously published data. |
| 67 | Efficacy of Indacaterol/Glycopyrronium in Patients with COPD Who Have Increased Dyspnea with Daily Activities | Mahler et al. | COPD (2016) | The intervention duration of this study was less than 48 weeks. |
| 68 | Efficacy and safety of umeclidinium/vilanterol 62.5/25 mcg and tiotropium 18 mcg in chronic obstructive pulmonary disease: results of a 24-week, randomized, controlled trial | Maleki-Yazdi et al. | Respiratory Medicine (2014) | This study was not designed as a parallel group RCT. |
| 69 | Effects of a combination of umeclidinium/vilanterol on exercise endurance in patients with chronic obstructive pulmonary disease: two randomized, double-blind clinical trials | Maltais et al. | Therapeutic Advances in Respiratory Disease (2014) | The intervention duration of this study was less than 48 weeks. |
| 70 | Effect of 12 weeks of once-daily tiotropium/olodaterol on exercise endurance during constant work-rate cycling and endurance shuttle walking in chronic obstructive pulmonary disease | Maltais et al. | Therapeutic Advances in Respiratory Disease (2018) | This study was not designed as a parallel group RCT. |
| 71 | Dual bronchodilation with tiotropium/ olodaterol further reduces activity-related breathlessness versus tiotropium alone in COPD | Maltais et al. | European Respiratory Journal (2019) | The intervention duration of this study was less than 48 weeks. |
| 72 | Dose Determination for a Fixed-Dose Drug Combination: A Phase II Randomized Controlled Trial for Tiotropium/Olodaterol Versus Tiotropium in Patients with COPD | Maltais et al. | Advances in Therapy (2019) | This study was eligible for our study, but we used another reference with the same data source. |
| 73 | Baseline Symptom Score Impact on Benefits of Glycopyrrolate/Formoterol Metered Dose Inhaler in COPD | Martinez et al. | Chest (2017) | The intervention duration of this study was less than 48 weeks. |
| 74 | Efficacy and Safety of Glycopyrrolate/Formoterol Metered Dose Inhaler Formulated Using Co-Suspension Delivery Technology in Patients With COPD | Martinez et al. | Chest (2017) | This study was a pooled analysis of previous RCTs. |
| 75 | The efficacy of aclidinium/formoterol on lung function and symptoms in patients with COPD categorized by symptom status: a pooled analysis | Miravitlles et al. | International Journal of Chronic Obstructive Pulmonary Disease (2016) | The intervention duration of this study was less than 48 weeks. |
| 76 | Effects of combined tiotropium/olodaterol on inspiratory capacity and exercise endurance in COPD | O’Donnell et al. | European Respiratory Journal (2017) | This reference did not describe the exacerbation rate or mortality rate, which were the outcomes of our meta-analysis. |
| 77 | Efficacy and safety of switch from tiotropium to indacaterol/glycopyrronium in symptomatic mildto-moderate COPD patients from Korea: A randomized, open-label, 12-week trial | Rhee et al.1 | Respirology (2018) (Abstract) | This study was not designed as a parallel group RCT. |
| 78 | Effects of umeclidinium/vilanterol on exercise endurance in COPD: a randomised study | Riley et al. | ERJ Open Research (2018) | The intervention duration of this study was less than 48 weeks. |
| 79 | AMPLIFY: a randomized, Phase III study evaluating the efficacy and safety of aclidinium/formoterol vs monocomponents and tiotropium in patients with moderate-to-very severe symptomatic COPD. | Sethi et al. | International Journal of Chronic Obstructive Pulmonary Disease (2019) | The intervention duration of this study was less than 48 weeks. |
| 80 | AMPLIFY: A randomized, phase III study evaluating the efficacy and safety of aclidinium/formoterol vs monocomponents and tiotropium in patients with moderate-to-very severe symptomatic COPD | Siler et al. | International Journal of Chronic Obstructive Pulmonary Disease (2016) | The intervention duration of this study was less than 48 weeks. |
| 81 | Efficacy and safety of aclidinium bromide/formoterol fumarate fixed-dose combinations compared with individual components and placebo in patients with COPD (ACLIFORM-COPD): a multicentre, randomised study | Singh et al. | BMC Pulmonary Medicine (2014) | The intervention duration of this study was less than 48 weeks. |
| 82 | Tiotropium + olodaterol shows clinically meaningful improvements in quality of life | Singh et al. | Respiratory Medicine (2015) | The intervention duration of this study was less than 48 weeks. |
| 83 | Umeclidinium/vilanterol versus fluticasone propionate/salmeterol in COPD: a randomised trial | Singh et al. | BMC Pulmonary Medicine (2015) | This study was eligible for our study, but we used another reference with the same data source. |
| 84 | Prevention of clinically important deteriorations in COPD with umeclidinium/vilanterol | Singh et al. | International Journal of Chronic Obstructive Pulmonary Disease (2016) | The study intervention did not meet the eligibility criteria of our study. |
| 85 | Single inhaler triple therapy versus inhaled corticosteroid plus long-acting beta2-agonist therapy for chronic obstructive pulmonary disease (TRILOGY): a double-blind, parallel group, randomised controlled trial | Singh et al. | Lancet (2016) | The study intervention did not meet the eligibility criteria of our study. |
| 86 | The bronchodilator effects of extrafine glycopyrronium added to combination treatment with beclometasone dipropionate plus formoterol in COPD: a randomised crossover study (the TRIDENT study) | Singh et al. | Respiratory Medicine (2016) | This study was a pooled analysis of previous RCTs |
| 87 | Reduction in clinically important deterioration in chronic obstructive pulmonary disease with aclidinium/formoterol | Singh et al. | Respiratory Research (2017) | This study was a post-hoc analysis using previously published data. |
| 88 | Relationship between exercise endurance and static hyperinflation in a post hoc analysis of two clinical trials in patients with COPD | Singh et al. | International Journal of Chronic Obstructive Pulmonary Disease (2018) | The intervention duration of this study was less than 48 weeks. |
| 89 | Formoterol and tiotropium compared with tiotropium alone for treatment of COPD | Tashkin et al. | COPD (2009) | This study was not designed as a parallel group RCT. |
| 90 | A multicenter, randomized, double-blind dose-ranging study of glycopyrrolate/formoterol fumarate fixed-dose combination metered dose inhaler compared to the monocomponents and open-label tiotropium dry powder inhaler in patients with moderate-to-severe COPD | Tashkin et al. | Respiratory Medicine (2016) | This study was eligible for our study, but we used another reference with the same data source. |
| 91 | Effect of 8 and 12 weeks' once-daily tiotropium and olodaterol, alone and combined with exercise training, on exercise endurance during walking in patients with copd | Troosters et al. | European Respiratory Journal (2016) (Abstract) | The intervention duration of this study was less than 48 weeks. |
| 92 | Effect of bronchodilation, exercise training, and behavior modification on symptoms and physical activity in chronic obstructive pulmonary disease | Troosters et al. | American Journal of Respiratory and Critical Care Medicine (2018) | This study was a pooled analysis of previous RCTs. |
| 93 | Response to Indacaterol/Glycopyrronium (IND/GLY) by Sex in Patients with COPD: a Pooled Analysis from the IGNITE Program | Tsiligianni et al. | COPD (2017) | This study was not designed as a parallel group RCT. |
| 94 | Combining tiotropium and salmeterol in COPD: Effects on airflow obstruction and symptoms | van Noord et al. | Respiratory Medicine (2010) | The intervention duration of this study was less than 48 weeks. |
| 95 | QVA149 demonstrates superior bronchodilation compared with indacaterol or placebo in patients with chronic obstructive pulmonary disease | van Noord et al. | Thorax (2010) | The intervention duration of this study was less than 48 weeks. |
| 96 | Efficacy and safety of coadministration of once-daily indacaterol and glycopyrronium versus indacaterol alone in COPD patients: the GLOW6 study | Vincken et al. | International Journal of Chronic Obstructive Pulmonary Disease (2014) | The intervention duration of this study was less than 48 weeks. |
| 97 | Formoterol mono- and combination therapy with tiotropium in patients with COPD: A 6-month study | Vogelmeier et al. | Respiratory Medicine (2008) | The intervention duration of this study was less than 48 weeks. |
| 98 | Efficacy and safety of once-daily QVA149 compared with twice-daily salmeterol-fluticasone in patients with chronic obstructive pulmonary disease (ILLUMINATE): A randomised, double-blind, parallel group study | Vogelmeier et al. | Lancet Respiratory Medicine (2013) | The intervention duration of this study was less than 48 weeks. |
| 99 | Efficacy and safety of aclidinium/formoterol versus salmeterol/fluticasone: a phase 3 COPD study | Vogelmeier et al. | European Respiratory Journal (2016) | The intervention duration of this study was less than 48 weeks. |
| 100 | Indacaterol/glycopyrronium in symptomatic patients with COPD (GOLD B and GOLD D) versus salmeterol/fluticasone: iLLUMINATE/LANTERN pooled analysis | Vogelmeier et al. | International Journal of Chronic Obstructive Pulmonary Disease (2016) | This study was a pooled analysis of previous RCTs. |
| 101 | Efficacy and safety of direct switch to indacaterol/glycopyrronium in patients with moderate COPD: the CRYSTAL open-label randomised trial | Vogelmeier et al. | Respiratory Research (2017) | The study intervention did not meet the eligibility criteria of our study. |
| 102 | Effects of indacaterol/glycopyrronium (QVA149) on lung hyperinflation and physical activity in patients with moderate to severe COPD: a randomised, placebo-controlled, crossover study (The MOVE Study) | Watz et al. | BMC Pulmonary Medicine (2016) | This study was not designed as a parallel group RCT. |
| 103 | ACTIVATE: the effect of aclidinium/formoterol on hyperinflation, exercise capacity, and physical activity in patients with COPD | Watz et al. | International Journal of Chronic Obstructive Pulmonary Disease (2017) | The intervention duration of this study was less than 48 weeks. |
| 104 | Pooled safety analysis of the fixed-dose combination of indacaterol and glycopyrronium (QVA149), its monocomponents, and tiotropium versus placebo in COPD patients | Wedzicha et al. | Respiratory Medicine (2014) | This study was a pooled analysis of previous RCTs. |
| 105 | Indacaterol/glycopyrronium versus salmeterol/fluticasone in Asian patients with COPD at a high risk of exacerbations: results from the FLAME study | Wedzicha et al. | International Journal of Chronic Obstructive Pulmonary Disease (2017) | This study was eligible for our study, but we used another reference with the same data source. |
| 106 | Efficacy and safety of once-daily inhaled umeclidinium/vilanterol in Asian patients with COPD: results from a randomized, placebo-controlled study | Zheng et al. | International Journal of Chronic Obstructive Pulmonary Disease (2015) | The intervention duration of this study was less than 48 weeks. |
| 107 | LANTERN: a randomized study of QVA149 versus salmeterol/fluticasone combination in patients with COPD | Zhong et al. | International Journal of Chronic Obstructive Pulmonary Disease (2015) | The intervention duration of this study was less than 48 weeks. |
| 108 | Efficacy and Safety of Indacaterol/Glycopyrronium (IND/GLY) Versus Salmeterol/Fluticasone in Chinese Patients with Moderate-to-Severe Chronic Obstructive Pulmonary Disease: the Chinese Cohort from the LANTERN Study | Zhong et al. | COPD (2016) | This study was eligible for our study, but we used another reference with the same data source. |
| 109 | Efficacy and safety of combining olodaterol Respimat and tiotropium HandiHaler in patients with COPD: results of two randomized, double-blind, active-controlled studies | ZuWallack et al. | International Journal of Chronic Obstructive Pulmonary Disease (2014) | The intervention duration of this study was less than 48 weeks. |
| 110 | Effects of Dual Bronchodilator Treatment on Cardiopulmonary Interactions in COPD | Unpublished | NCT03425617 | This reference did not describe the exacerbation rate or mortality rate, which were the outcomes of our meta-analysis |
| 111 | Bronchodilator Effect of RPL554 Administered in Addition to Tiotropium/Olodaterol in Patients With COPD | Unpulbished | NCT03673670 | This study was not designed as a parallel group RCT. |
