# Supplementary material for: Comparisons of exacerbations and mortality among LAMA/LABA combinations in stable chronic obstructive pulmonary disease: systematic review and Bayesian network meta-analysis
Source: Respir Res. 2020 Nov 25;21:310. doi: 10.1186/s12931-020-01540-8 (PMC7687787; doi:10.1186/s12931-020-01540-8)

**Additional file 4. The Cochrane Collaboration’s tool for assessing risk of bias for included randomized controlled trials**

| **Author (year)**  **Study name** | **Domain** | **Review author’s assessment and reason for judgment** |
| --- | --- | --- |
| **Aaron et al. (2007)** | Random sequence generation | Low risk of bias. Random sequence was prepared from a computer-generated random listing of the three treatment allocations. |
|  | Allocation concealment | Low risk of bias. Randomization was performed using central allocation of a randomization schedule and research staff were not aware of the treatment assignment. |
|  | Blinding of participants and personnel | Low risk of bias. Neither research staff nor patients were aware of the treatment assignment before or after randomization. |
|  | Blinding of outcome assessment | Low risk of bias. The specific method of blinding of outcome assessment was not described. However, since our primary and secondary outcomes were objective findings (acute exacerbation and mortality rate), it is judged that the outcome was not likely to be influenced by the lack of blinding of outcome assessment. |
|  | Incomplete outcome data | Low risk of bias. Patients in both groups were followed all the way through to the end of the study. As missing data was balanced between two arms, withdrawal or follow-up loss in surrogates would not have influenced our primary and secondary outcomes. |
|  | Selective reporting | Low risk of bias. A pre-existing protocol was described and all the results were reported in the pre-specified manner. |
|  | Other sources of bias | Low risk of bias. This study appears to be free of other sources of bias. |
| **Asai et al. (2013)**  **ARISE** | Random sequence generation | Unclear risk of bias. The specific method of random sequence generation was not described. |
|  | Allocation concealment | Unclear risk of bias. The specific method of allocation concealment from participants and medical personnel was not described. |
|  | Blinding of participants and personnel | High risk of bias. Open-label tiotropium hand inhaler was used in control arm, and placebo use was not reported in either arm. |
|  | Blinding of outcome assessment | Low risk of bias. The specific method of blinding of outcome assessment was not described. However, since our primary outcome was an objective finding (acute exacerbation and mortality), the outcome was unlikely to have been influenced by the lack of blinding. |
|  | Incomplete outcome data | Low risk of bias. Withdrawal or follow-up loss rates in eligible patients were minimal (3%) and almost balanced between the two comparison groups. |
|  | Selective reporting | Unclear risk of bias. A pre-existing protocol was described, but no records showing full data were available. Only the abstract was found. |
|  | Other sources of bias | Low risk of bias. This study appears to be free of other sources of bias. |
| **Dahl et al. (2013)**  **ENLIGHTEN** | Random sequence generation | Unclear risk of bias. The specific method of random sequence generation was not described. |
|  | Allocation concealment | Unclear risk of bias. The specific method of allocation concealment from participants and medical personnel was not described. |
|  | Blinding of participants and personnel | Low risk of bias. Patients in the intervention and control arms (placebo) used same inhaler device (Breezhaler) |
|  | Blinding of outcome assessment | Low risk of bias. The specific method of blinding of outcome assessment was not described. However, since our primary outcome was an objective finding (acute exacerbation and mortality), the outcome was unlikely to have been influenced by the lack of blinding. |
|  | Incomplete outcome data | Low risk of bias. About 16.5% of randomized patients were discontinued, but the reasons for withdrawal were almost balanced. |
|  | Selective reporting | Low risk of bias. A pre-existing protocol was described, and all results were reported in the pre-specified manner. |
|  | Other sources of bias | Low risk of bias. This study appears to be free of other sources of bias. |
| **Wedzicha et al. (2013)**  **SPARK** | Random sequence generation | Low risk of bias. Investigators contacted an interactive voice response system or web system, which generated a randomization number (not communicated to the caller) that linked the patient to a treatment group. |
|  | Allocation concealment | Low risk of bias. Patients were randomly allocated to each treatment group. Patients randomly allocated to open-label tiotropium were not assigned a medication number because this treatment was supplied locally. |
|  | Blinding of participants and personnel | Low risk of bias. Patients, investigative staff, and people performing assessments were masked to treatment (QVA149 or glycopyrronium) and from randomization until database lock. Although open-label tiotropium was used, all the double-blind study drugs were identical in packaging, labelling, schedule of administration, appearance, taste, and odor. |
|  | Blinding of outcome assessment | Low risk of bias. Data analysts were masked to treatment (QVA149 or glycopyrronium) and from randomization until database lock. Unmasking occurred in the case of emergencies and at the conclusion of the study. |
|  | Incomplete outcome data | Unclear risk of bias. Withdrawal rate was 24.7%–27.4% and relatively even among arms. Primary analysis was done without imputation. Sensitivity analysis according to imputation was done, but imputation method is not clearly described. |
|  | Selective reporting | Low risk of bias. A pre-existing protocol was described and all the results were reported in the pre-specified manner. |
|  | Other sources of bias | Low risk of bias. This study appears to be free of other sources of bias. |
| **Donohue et al (2014)** | Random sequence generation | Low risk of bias. Randomization was carried out by assigning patient identification numbers via an interactive web response system. |
|  | Allocation concealment | Low risk of bias. Although the specific method of allocation concealment from participants and medical personnel was not described, researchers stated that the randomization was conducted using an interactive web response system, in which unblinding was only allowed in emergencies. |
|  | Blinding of participants and personnel | Low risk of bias. Each group used the same inhalers in the same manner. |
|  | Blinding of outcome assessment | Low risk of bias. The specific method of blinding of outcome assessment was not described. However, since our primary outcome was an objective finding (acute exacerbation and mortality), the outcome was unlikely to have been influenced by the lack of blinding. |
|  | Incomplete outcome data | Low risk of bias. Withdrawal rate was 36.7%-41.4%, but relatively even among arms. As our study extracted data from intention-to-treat participants, there was no impact on our outcomes. |
|  | Selective reporting | Low risk of bias. A pre-existing protocol was described. Although there were additional post-hoc analyses of adverse events, the authors clearly described these in the article, and adverse events were not our analysis target. |
|  | Other sources of bias | Low risk of bias. This study appears to be free of other sources of bias. |
| **Buhl et al. (2015)**  **TONADO I** | Random sequence generation | Low risk of bias. Treatment was assigned via an interactive voice response system / interactive web response system. |
|  | Allocation concealment | Low risk of bias. Although the specific method of allocation concealment from participants and medical personnel was not described, researchers stated that the randomization was conducted using an interactive web response system, in which unblinding was only allowed in emergencies. |
|  | Blinding of participants and personnel | Low risk of bias. Each group used the same inhalers in the same manner. |
|  | Blinding of outcome assessment | Low risk of bias. An independent data monitoring committee regularly reviewed unblinded safety data. |
|  | Incomplete outcome data | Low risk of bias. Withdrawal rate was 13.8% and relatively even among arms. As our study extracted data from intention-to-treat participants, there was no impact on our outcomes. |
|  | Selective reporting | Low risk of bias. A pre-existing protocol was described and all the results were reported in the pre-specified manner. |
|  | Other sources of bias | Low risk of bias. This study appears to be free of other sources of bias. |
| **Buhl et al. (2015)**  **TONADO II** | Random sequence generation | Low risk of bias. Treatment was assigned via an interactive voice response system / interactive web response system. |
|  | Allocation concealment | Low risk of bias. Although the specific method of allocation concealment from participants and medical personnel was not described, researchers stated that the randomization was conducted using an interactive web response system, in which unblinding was only allowed in emergencies. |
|  | Blinding of participants and personnel | Low risk of bias. Each group used the same inhalers in the same manner. |
|  | Blinding of outcome assessment | Low risk of bias. An independent data monitoring committee regularly reviewed unblinded safety data. |
|  | Incomplete outcome data | Low risk of bias. The withdrawal rate was 13.8% and relatively even among arms. As our study extracted data from intention-to-treat participants, there was no impact on our outcomes. |
|  | Selective reporting | Low risk of bias. A pre-existing protocol was described and all the results were reported in the pre-specified manner. |
|  | Other sources of bias | Low risk of bias. This study appears to be free of other sources of bias. |
| **Larbig et al. (2015)**  **RADIATE** | Random sequence generation | Unclear risk of bias. In both the abstract and clinicaltrial.gov, the specific method of random sequence generation was not described. |
|  | Allocation concealment | Unclear risk of bias. In both the abstract and clinicaltrial.gov, the specific method of allocation concealment from participants and medical personnel was not described. |
|  | Blinding of participants and personnel | Unclear risk of bias. In both the abstract and clinicaltrial.gov, the specific method of blinding of participants and medical personnel was not described. |
|  | Blinding of outcome assessment | Low risk of bias. The specific method of blinding of outcome assessment was not described. However, since our primary outcome was an objective finding (acute exacerbation and mortality), the outcome was unlikely to have been influenced by the lack of blinding. |
|  | Incomplete outcome data | Low risk of bias. The number of participants withdrawn after randomization was not described in either the abstract or clinicaltrial.gov. However, as we extracted data from intention-to-treat participants, there was no influence on our meta-analysis. |
|  | Selective reporting | Unclear risk of bias. A pre-existing protocol was described, but no records showing full data were available. Only an abstract was found. |
|  | Other sources of bias | Low risk of bias. This study appears to be free of other sources of bias. |
| **Donohue et al. (2016)** | Random sequence generation | Low risk of bias. Randomization was carried out by assigning patient identification numbers via an interactive web response system. |
|  | Allocation concealment | Low risk of bias. An interactive web response system is typically used to maintain allocation concealment by having a third party conduct randomization. As this study used an interactive web response system for randomization, allocation concealment was conducted properly. |
|  | Blinding of participants and personnel | Low risk of bias. Each group used the same inhalers in the same manner. |
|  | Blinding of outcome assessment | Low risk of bias. The specific method of blinding of outcome assessment was not described. However, since our primary outcome was an objective finding (acute exacerbation and mortality), the outcome was unlikely to have been influenced by the lack of blinding. |
|  | Incomplete outcome data | Low risk of bias. Withdrawal rate was 32.4%–32.8%, but relatively even among arms. As our study extracted data from intention-to-treat participants, there was no impact on our outcomes. |
|  | Selective reporting | Low risk of bias. A pre-existing protocol was described and all the results were reported in the pre-specified manner. |
|  | Other sources of bias | Low risk of bias. This study appears to be free of other sources of bias. |
| **Ferguson et al. (2016)**  **FLIGHT3** | Random sequence generation | Low risk of bias. All eligible patients were randomized using interactive response technology to one of the treatment groups. |
|  | Allocation concealment | Low risk of bias. Interactive response technology is typically used to maintain allocation concealment by having a third party conduct randomization. As this study used an interactive web response system for randomization, allocation concealment was conducted properly. |
|  | Blinding of participants and personnel | Low risk of bias. Patients were instructed to take one capsule each morning and one in the evening. Each group used the same inhalers in the same manner. |
|  | Blinding of outcome assessment | Low risk of bias. The specific method of blinding of outcome assessment was not described. However, since our primary outcome was an objective finding (acute exacerbation and mortality), the outcome was unlikely to have been influenced by the lack of blinding. |
|  | Incomplete outcome data | Low risk of bias. Withdrawal rate was 8.3%–13.2%, and relatively even among arms. As our study extracted data from intention-to-treat participants, there was no impact on our outcomes. |
|  | Selective reporting | Low risk of bias. A pre-existing protocol was described and all the results were reported in the pre-specified manner. |
|  | Other sources of bias | Low risk of bias. This study appears to be free of other sources of bias. |
| **Wedzicha et al. (2016)**  **FLAME** | Random sequence generation | Low risk of bias. Interactive response technology assigned each patient a randomization number, which was used to link the patient to a treatment arm and specified a unique medication number for the first package of investigational treatment to be dispensed to the patient. |
|  | Allocation concealment | Low risk of bias. Investigational treatment identity was concealed using identical packaging, labeling, schedule of administration, and appearance. |
|  | Blinding of participants and personnel | Low risk of bias. Patients, investigator staff, persons performing the assessments, and data analysts remained blind to the identity of the treatment from randomization until database lock. |
|  | Blinding of outcome assessment | Low risk of bias. Randomization data were kept strictly confidential until unblinding, and were inaccessible to anyone involved in the study |
|  | Incomplete outcome data | Low risk of bias. More than 99.7% of patients were included for intention-to-treat analysis. |
|  | Selective reporting | Low risk of bias. A pre-existing protocol was prospectively registered and well reported with additional online supplemental material. |
|  | Other sources of bias | Low risk of bias. This study appears to be free of other sources of bias. |
| **D'Urzo et al. (2017)**  **AUGMENT** | Random sequence generation | Low risk of bias. A list of patient randomization codes was generated using statistical programming at Forest Research Institute. |
|  | Allocation concealment | Low risk of bias. As allocation was implemented using an interactive web response system, allocation concealment was conducted properly. |
|  | Blinding of participants and personnel | Low risk of bias. All inhalers were identical from an external perspective and the only difference between  them was that the placebo did not contain active ingredient. |
|  | Blinding of outcome assessment | Low risk of bias. The specific method of blinding of outcome assessment was not described. However, since our primary outcome was an objective finding (acute exacerbation and mortality), the outcome was unlikely to have been influenced by the lack of blinding. |
|  | Incomplete outcome data | High risk of bias. There was a significant amount of incomplete outcome data. Among the 1322 participants who completed the lead-in study, 921 were enrolled for the extension study. There was no information about the missing participants from the lead-in study. |
|  | Selective reporting | Low risk of bias. A pre-existing protocol was described and all the results were reported in the pre-specified manner. |
|  | Other sources of bias | High risk of bias. The study design was defective in that it was not originally planned for 52 weeks. A lead-in study was conducted for 24 weeks and a further extension study for 28 weeks. |
| **Hanania et al. (2017)**  **PINNACLE-3** | Random sequence generation | Low risk of bias. Study participants were randomly assigned for double-blinded treatment using a centralized interactive web response system. |
|  | Allocation concealment | Low risk of bias. Interactive response technology is typically used to maintain allocation concealment by conducting randomization by a third party. As this study used interactive web response system for randomization, allocation concealment is considered conducted properly. |
|  | Blinding of participants and personnel | High risk of bias. This study used open-label tiotropium. Performance bias cannot be excluded. |
|  | Blinding of outcome assessment | Low risk of bias. The specific method of blinding of outcome assessment was not described. However, since our primary outcome was an objective finding (acute exacerbation and mortality), the outcome was unlikely to have been influenced by the lack of blinding. |
|  | Incomplete outcome data | High risk of bias. There was a significant amount of incomplete outcome data. Among the 2678 participants who completed the PINNACLE-1 and -2 studies, 892 were enrolled in the extension study. Subjects receiving placebo MDI were not included in the PINNACLE-3 study. Otherwise, there was no information about the missing participants from the PINNACLE-1 and -2 study. |
|  | Selective reporting | Low risk of bias. A pre-existing protocol was described and all the results were reported in the pre-specified manner. |
|  | Other sources of bias | High risk of bias. The study design was defective in that it was not originally planned for 52 weeks. PINNACLE-3 was a 28-week extension study of PINNACLE-1 and -2, the two pivotal 24-week studies. |
| **Ichinose et al. (2017)** | Random sequence generation | Low risk of bias. The randomization list was generated using a validated system, which involved a pseudo-random number generator. |
|  | Allocation concealment | Low risk of bias. Randomization to treatment was performed by a third party using an interactive voice/web-based response system, with medication boxes assigned to patients according to the unique number they were allocated. |
|  | Blinding of participants and personnel | Low risk of bias. Each group used the same inhalers in the same manner (Respimat inhaler; 2 puffs every morning). |
|  | Blinding of outcome assessment | Low risk of bias. The specific method of blinding of outcome assessment was not described. However, since our primary outcome was an objective finding (acute exacerbation and mortality), the outcome was unlikely to have been influenced by the lack of blinding. |
|  | Incomplete outcome data | Low risk of bias. The withdrawal rate was 4.5%–19.5%, and different among the arms. Most differences occurred in acute exacerbations of COPD, which were all included in the intention-to-treat analysis. For that reason, different withdrawal rates had no impact on our outcomes. |
|  | Selective reporting | Low risk of bias. A pre-existing protocol was described and all the results were reported in the pre-specified manner. |
|  | Other sources of bias | Low risk of bias. This study appears to be free of other sources of bias. |
| **Calverley et al. (2018)**  **DYNAGITO** | Random sequence generation | Low risk of bias. A random sequence was generated using validated randomization software by Boehringer Ingelheim. |
|  | Allocation concealment | Low risk of bias. An interactive response technology system was used for allocation concealment of trial medication. |
|  | Blinding of participants and personnel | Low risk of bias. Treatment was masked to patients and investigators, because treatments were delivered using identical Respimat devices. |
|  | Blinding of outcome assessment | Low risk of bias. By using identical Respimat devices, everyone involved in analyzing the trial data were blinded to the outcome assessment. |
|  | Incomplete outcome data | Low risk of bias. The withdrawal rate was 12.4%–16.5% and relatively even among arms. As our study extracted data from intention-to-treat participants, there was no impact on our outcomes. |
|  | Selective reporting | Low risk of bias. A pre-existing protocol was described and all the results were reported in the pre-specified manner. |
|  | Other sources of bias | Low risk of bias. This study appears to be free of other sources of bias. |
| **Lipson et al. (2018)**  **IMPACT** | Random sequence generation | Low risk of bias. A randomization code was generated using a validated computerized system. |
|  | Allocation concealment | Low risk of bias. The study used site-based randomization to allocate treatments using an interactive voice response system to maintain allocation concealment. |
|  | Blinding of participants and personnel | Low risk of bias. As the supplied DPIs were identical in appearance, neither the subject nor the investigator knew which one the subject was receiving. |
|  | Blinding of outcome assessment | Low risk of bias. The specific method of blinding of outcome assessment was not described. However, since our primary outcome was an objective finding (acute exacerbation and mortality), the outcome was unlikely to have been influenced by the lack of blinding. |
|  | Incomplete outcome data | Low risk of bias. The withdrawal rate was 18%–25% and relatively even among arms. As our study extracted data from intention-to-treat participants, there was no impact on our outcomes. |
|  | Selective reporting | Low risk of bias. A pre-existing protocol was described and all the results were reported in the pre-specified manner. |
|  | Other sources of bias | Low risk of bias. This study appears to be free of other sources of bias. |
| **Papi et al. (2018)**  **TRIBUTE** | Random sequence generation | Low risk of bias. A randomization list was generated by the interactive response technology provider. |
|  | Allocation concealment | Low risk of bias. Patients were randomly assigned to treatment groups by central randomization for allocation concealment. |
|  | Blinding of participants and personnel | Low risk of bias. Using a double-dummy approach, patients and investigators were masked to treatment assignment for the duration of the study. |
|  | Blinding of outcome assessment | Low risk of bias. The specific method of blinding of outcome assessment was not described. However, since our primary outcome was an objective finding (acute exacerbation and mortality), the outcome was unlikely to have been influenced by the lack of blinding. |
|  | Incomplete outcome data | Low risk of bias. The withdrawal rate was 12.8%–15.6% and relatively even among arms. As our study extracted data from intention-to-treat participants, there was no impact on our outcomes. |
|  | Selective reporting | Low risk of bias. A pre-existing protocol was described and all the results were reported in the pre-specified manner. |
|  | Other sources of bias | Low risk of bias. This study appears to be free of other sources of bias. |


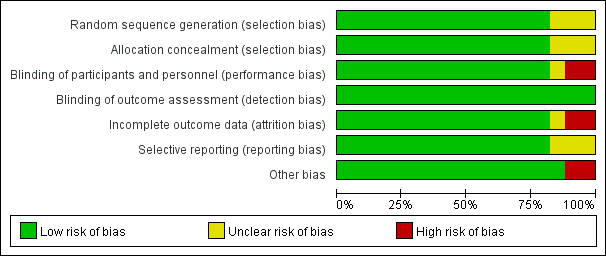


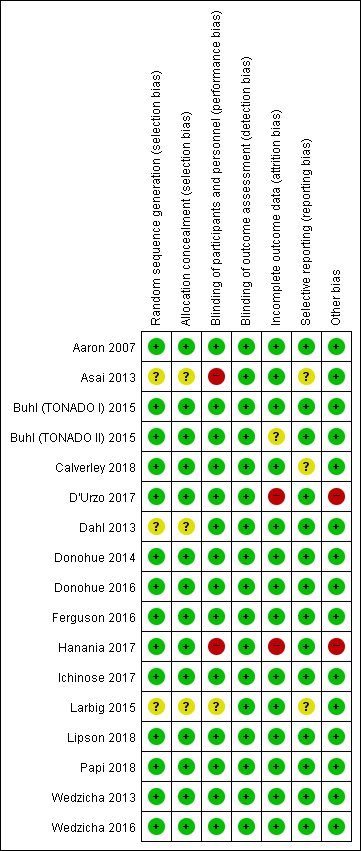

Supplement: Supplementary file 4 — Additional file 4. The Cochrane Collaboration’s tool for assessing risk of bias for included randomized controlled trials. [file 12931_2020_1540_MOESM4_ESM.docx]
