# Supplementary material for: Comparisons of exacerbations and mortality among LAMA/LABA combinations in stable chronic obstructive pulmonary disease: systematic review and Bayesian network meta-analysis
Source: Respir Res. 2020 Nov 25;21:310. doi: 10.1186/s12931-020-01540-8 (PMC7687787; doi:10.1186/s12931-020-01540-8)
Supplement: Supplementary file 5 — Additional file 5. Assessment of publication bias in the direct comparisons including 3 or more studies. [file 12931_2020_1540_MOESM5_ESM.docx]

**Additional file 5. Assessment of publication bias in the direct comparisons including 3 or more studies**

| Direct comparison | P-value of Egger test |
| --- | --- |
| Total exacerbation |  |
| Tiotropium vs. Glycopyrrolate/Indacaterol | 0.342 |
| All-cause mortality |  |
| Tiotropium vs. Glycopyrrolate/Indacaterol | 0.599 |
| Pneumonia |  |
| Tiotropium vs. Glycopyrrolate/Indacaterol | 0.409 |

ICS: inhaled corticosteroid, LABA: long-acting beta-agonist, LAMA: long-acting muscarinic antagonist, OR: odds ratio
