# Supplementary material for: Comparisons of exacerbations and mortality among LAMA/LABA combinations in stable chronic obstructive pulmonary disease: systematic review and Bayesian network meta-analysis
Source: Respir Res. 2020 Nov 25;21:310. doi: 10.1186/s12931-020-01540-8 (PMC7687787; doi:10.1186/s12931-020-01540-8)
Supplement: Supplementary file 6 — Additional file 6. Estimates of effects and quality ratings for comparison of LAMA/LABA from Bayesian network meta-analyses. [file 12931_2020_1540_MOESM6_ESM.docx]

**Additional file 6. Estimates of effects and quality ratings for comparison of LAMA/LABA from Bayesian network meta-analyses**

| Treatment | Comparator | Posterior median OR (95% CrIs) | Quality of evidence |
| --- | --- | --- | --- |
| Network A | | | |
| Total exacerbation | | | |
| Aclidinium / Formoterol | Tiotropium / Olodaterol | 0.99 (0.60-1.60) | Low*,‡ |
| Umeclidinium / Vilanterol | Tiotropium / Olodaterol | 0.42 (0.19-0.97) | Moderate‡ |
| Glycopyrrolate / Formoterol | Tiotropium / Olodaterol | 0.92 (0.61-1.41) | Moderate* |
| Glycopyrrolate / Indacaterol | Tiotropium / Olodaterol | 1.02 (0.74-1.45) | Moderate* |
| Tiotropium / Salmeterol | Tiotropium / Olodaterol | 1.12 (0.62-2.06) | High |
| Umeclidinium / Vilanterol | Aclidinium / Formoterol | 0.43 (0.20-0.94) | Moderate* |
| Glycopyrrolate / Formoterol | Aclidinium / Formoterol | 0.94 (0.59-1.50) | Moderate* |
| Glycopyrrolate / Indacaterol | Aclidinium / Formoterol | 1.04 (0.68-1.66) | Low*,‡ |
| Tiotropium / Salmeterol | Aclidinium / Formoterol | 1.14 (0.56-2.34) | Low*,‡ |
| Glycopyrrolate / Formoterol | Umeclidinium / Vilanterol | 2.18 (0.95-4.93) | Low*,‡ |
| Glycopyrrolate / Indacaterol | Umeclidinium / Vilanterol | 2.43 (1.10-5.36) | Moderate* |
| Tiotropium / Salmeterol | Umeclidinium / Vilanterol | 2.66 (1.00-7.09) | Low‡,§ |
| Glycopyrrolate / Indacaterol | Glycopyrrolate / Formoterol | 1.11 (0.76-1.66) | Moderate* |
| Tiotropium / Salmeterol | Glycopyrrolate / Formoterol | 1.22 (0.62-2.37) | Moderate* |
| Tiotropium / Salmeterol | Glycopyrrolate / Indacaterol | 1.09 (0.59-2.02) | Moderate* |
| Moderate to severe exacerbation | | | |
| Aclidinium / Formoterol | Tiotropium / Olodaterol | 0.95 (0.18-5.04) | Low*,‡ |
| Umeclidinium / Vilanterol | Tiotropium / Olodaterol | 1.05 (0-91490) | Very low‡,§§ |
| Glycopyrrolate / Formoterol | Tiotropium / Olodaterol | 0.9 (0.22-4.16) | Moderate* |
| Glycopyrrolate / Indacaterol | Tiotropium / Olodaterol | 1.08 (0.26-4.86) | Moderate* |
| Tiotropium / Salmeterol | Tiotropium / Olodaterol | Not estimable | - |
| Umeclidinium / Vilanterol | Aclidinium / Formoterol | 1.09 (0-97430) | Very low*,§§ |
| Glycopyrrolate / Formoterol | Aclidinium / Formoterol | 0.95 (0.26-3.69) | Moderate* |
| Glycopyrrolate / Indacaterol | Aclidinium / Formoterol | 1.13 (0.25-5.38) | Low*,‡ |
| Tiotropium / Salmeterol | Aclidinium / Formoterol | Not estimable | - |
| Glycopyrrolate / Formoterol | Umeclidinium / Vilanterol | 0.86 (0-68860) | Very low*,‡,§§ |
| Glycopyrrolate / Indacaterol | Umeclidinium / Vilanterol | 1.04 (0-80330) | Very low*,§§ |
| Tiotropium / Salmeterol | Umeclidinium / Vilanterol | Not estimable | - |
| Glycopyrrolate / Indacaterol | Glycopyrrolate / Formoterol | 1.19 (0.35-4.09) | Moderate* |
| Tiotropium / Salmeterol | Glycopyrrolate / Formoterol | Not estimable | - |
| Tiotropium / Salmeterol | Glycopyrrolate / Indacaterol | Not estimable | - |
| All-cause mortality | | | |
| Aclidinium / Formoterol | Tiotropium / Olodaterol | 0.9 (0.05-21.92) | Very low*,‡,§ |
| Umeclidinium / Vilanterol | Tiotropium / Olodaterol | 0.03 (0-7.15) | Low‡,§ |
| Glycopyrrolate / Formoterol | Tiotropium / Olodaterol | 0.74 (0.05-14.51) | Low*,§ |
| Glycopyrrolate / Indacaterol | Tiotropium / Olodaterol | 1.05 (0.17-8.7) | Moderate* |
| Tiotropium / Salmeterol | Tiotropium / Olodaterol | 1.68 (0.1-27.64) | Moderate§ |
| Umeclidinium / Vilanterol | Aclidinium / Formoterol | 0.03 (0-6.88) | Low*,§ |
| Glycopyrrolate / Formoterol | Aclidinium / Formoterol | 0.82 (0.03-19.07) | Low*,§ |
| Glycopyrrolate / Indacaterol | Aclidinium / Formoterol | 1.19 (0.07-18.77) | Very low*,‡,§ |
| Tiotropium / Salmeterol | Aclidinium / Formoterol | 1.87 (0.04-59.48) | Very low*,‡,§ |
| Glycopyrrolate / Formoterol | Umeclidinium / Vilanterol | 29.98 (0.09-41250) | Very low*,‡,§§ |
| Glycopyrrolate / Indacaterol | Umeclidinium / Vilanterol | 42.73 (0.18-46040) | Very low*,§§ |
| Tiotropium / Salmeterol | Umeclidinium / Vilanterol | 68.98 (0.16-92860) | Very low‡,§§ |
| Glycopyrrolate / Indacaterol | Glycopyrrolate / Formoterol | 1.43 (0.09-21.95) | Low*,§ |
| Tiotropium / Salmeterol | Glycopyrrolate / Formoterol | 2.3 (0.06-59.11) | Low*,§ |
| Tiotropium / Salmeterol | Glycopyrrolate / Indacaterol | 1.57 (0.08-22.96) | Low*,§ |
| COPD-related mortality | | | |
| Aclidinium / Formoterol | Tiotropium / Olodaterol | 0.01 (0-1439) | Very low*,‡,§§ |
| Umeclidinium / Vilanterol | Tiotropium / Olodaterol | 1.93 (0-237100) | Very low‡,§§ |
| Glycopyrrolate / Formoterol | Tiotropium / Olodaterol | 0.05 (0-75270) | Very low*,§§ |
| Glycopyrrolate / Indacaterol | Tiotropium / Olodaterol | 41.3 (0-10600000) | Very low*,§§ |
| Tiotropium / Salmeterol | Tiotropium / Olodaterol | Not estimable | - |
| Umeclidinium / Vilanterol | Aclidinium / Formoterol | 274 (0-7690000000) | Very low*,§§ |
| Glycopyrrolate / Formoterol | Aclidinium / Formoterol | 6.77 (0-858000000) | Very low*,§§ |
| Glycopyrrolate / Indacaterol | Aclidinium / Formoterol | 6241 (0-386000000000) | Very low*,‡,§§ |
| Tiotropium / Salmeterol | Aclidinium / Formoterol | Not estimable | - |
| Glycopyrrolate / Formoterol | Umeclidinium / Vilanterol | 0.02 (0-1910000) | Very low*,‡,§§ |
| Glycopyrrolate / Indacaterol | Umeclidinium / Vilanterol | 22.16 (0-451000000) | Very low*,§§ |
| Tiotropium / Salmeterol | Umeclidinium / Vilanterol | Not estimable | - |
| Glycopyrrolate / Indacaterol | Glycopyrrolate / Formoterol | 1015 (0-2200000000000) | Very low*,§§ |
| Tiotropium / Salmeterol | Glycopyrrolate / Formoterol | Not estimable | - |
| Tiotropium / Salmeterol | Glycopyrrolate / Indacaterol | Not estimable | - |
| Cardiovascular disease-related mortality | | | |
| Aclidinium / Formoterol | Tiotropium / Olodaterol | 0.04 (0-34) | Very low*,‡,§ |
| Umeclidinium / Vilanterol | Tiotropium / Olodaterol | 0.34 (0-11300) | Very low‡,§§ |
| Glycopyrrolate / Formoterol | Tiotropium / Olodaterol | 1.43 (0.02-244) | Very low*,§§ |
| Glycopyrrolate / Indacaterol | Tiotropium / Olodaterol | 1.19 (0-1010000) | Very low*,§§ |
| Tiotropium / Salmeterol | Tiotropium / Olodaterol | Not estimable | - |
| Umeclidinium / Vilanterol | Aclidinium / Formoterol | 9.08 (0-2050000) | Very low*,§§ |
| Glycopyrrolate / Formoterol | Aclidinium / Formoterol | 33.7 (0.08-125700) | Very low*,§§ |
| Glycopyrrolate / Indacaterol | Aclidinium / Formoterol | 31 (0-134000000) | Very low*,‡,§§ |
| Tiotropium / Salmeterol | Aclidinium / Formoterol | Not estimable | - |
| Glycopyrrolate / Formoterol | Umeclidinium / Vilanterol | 4.6 (0-158000) | Very low*,‡,§§ |
| Glycopyrrolate / Indacaterol | Umeclidinium / Vilanterol | 3.7 (0-41900000) | Very low*,§§ |
| Tiotropium / Salmeterol | Umeclidinium / Vilanterol | Not estimable | - |
| Glycopyrrolate / Indacaterol | Glycopyrrolate / Formoterol | 0.77 (0-912000) | Very low*,§§ |
| Tiotropium / Salmeterol | Glycopyrrolate / Formoterol | Not estimable | - |
| Tiotropium / Salmeterol | Glycopyrrolate / Indacaterol | Not estimable | - |
| Major adverse cardiac events | | | |
| Aclidinium / Formoterol | Tiotropium / Olodaterol | 0.24 (0.01-8.83) | Very low*,‡,§ |
| Umeclidinium / Vilanterol | Tiotropium / Olodaterol | Not estimable | - |
| Glycopyrrolate / Formoterol | Tiotropium / Olodaterol | 0.4 (0.01-11.56) | Low*,§ |
| Glycopyrrolate / Indacaterol | Tiotropium / Olodaterol | 1.94 (0.09-88.14) | Very low*,†,§ |
| Tiotropium / Salmeterol | Tiotropium / Olodaterol | Not estimable | - |
| Umeclidinium / Vilanterol | Aclidinium / Formoterol | Not estimable | - |
| Glycopyrrolate / Formoterol | Aclidinium / Formoterol | 1.64 (0.08-40.72) | Low*,§ |
| Glycopyrrolate / Indacaterol | Aclidinium / Formoterol | 8.06 (0.35-443) | Very low*,†,‡,§§ |
| Tiotropium / Salmeterol | Aclidinium / Formoterol | Not estimable | - |
| Glycopyrrolate / Formoterol | Umeclidinium / Vilanterol | Not estimable | - |
| Glycopyrrolate / Indacaterol | Umeclidinium / Vilanterol | Not estimable | - |
| Tiotropium / Salmeterol | Umeclidinium / Vilanterol | Not estimable | - |
| Glycopyrrolate / Indacaterol | Glycopyrrolate / Formoterol | 4.88 (0.24-190.6) | Very low*,†,§§ |
| Tiotropium / Salmeterol | Glycopyrrolate / Formoterol | Not estimable | - |
| Tiotropium / Salmeterol | Glycopyrrolate / Indacaterol | Not estimable | - |
| Pneumonia | | | |
| Aclidinium / Formoterol | Tiotropium / Olodaterol | 0.85 (0.13-5.22) | Low*,‡ |
| Umeclidinium / Vilanterol | Tiotropium / Olodaterol | 0.65 (0.04-11.25) | Low‡,§ |
| Glycopyrrolate / Formoterol | Tiotropium / Olodaterol | 1.26 (0.32-5.38) | Moderate* |
| Glycopyrrolate / Indacaterol | Tiotropium / Olodaterol | 1.17 (0.46-4.38) | Low*,† |
| Tiotropium / Salmeterol | Tiotropium / Olodaterol | 24.85 (0.13-164500) | Low§§ |
| Umeclidinium / Vilanterol | Aclidinium / Formoterol | 0.75 (0.05-12.24) | Low*,§ |
| Glycopyrrolate / Formoterol | Aclidinium / Formoterol | 1.49 (0.25-9.62) | Moderate* |
| Glycopyrrolate / Indacaterol | Aclidinium / Formoterol | 1.4 (0.27-9.69) | Low*,‡ |
| Tiotropium / Salmeterol | Aclidinium / Formoterol | 30.82 (0.12-224300) | Very low*,‡,§§ |
| Glycopyrrolate / Formoterol | Umeclidinium / Vilanterol | 1.94 (0.11-33.85) | Low*,‡ |
| Glycopyrrolate / Indacaterol | Umeclidinium / Vilanterol | 1.84 (0.12-32.73) | Moderate* |
| Tiotropium / Salmeterol | Umeclidinium / Vilanterol | 42.81 (0.09-341300) | Very low‡,§§ |
| Glycopyrrolate / Indacaterol | Glycopyrrolate / Formoterol | 0.93 (0.29-4.04) | Low*,† |
| Tiotropium / Salmeterol | Glycopyrrolate / Formoterol | 19.79 (0.1-137800) | Very low*,§§ |
| Tiotropium / Salmeterol | Glycopyrrolate / Indacaterol | 20.54 (0.11-132100) | Very low*,†,§§ |
| Network B | | | |
| Total exacerbation | | | |
| Aclidinium / Formoterol | Tiotropium / Olodaterol | 1.14 (0.79-1.62) | Low*,‡ |
| Umeclidinium / Vilanterol | Tiotropium / Olodaterol | 1.06 (0.68-1.50) | Low‡ |
| Glycopyrrolate / Formoterol | Tiotropium / Olodaterol | 0.98 (0.67-1.45) | Low*,‡ |
| Glycopyrrolate / Indacaterol | Tiotropium / Olodaterol | 1.00 (0.76-1.34) | Low *,‡ |
| Tiotropium / Salmeterol | Tiotropium / Olodaterol | 1.17 (0.67-2.02) | Moderate,‡ |
| Umeclidinium / Vilanterol | Aclidinium / Formoterol | 0.92 (0.58-1.39) | Low*,‡ |
| Glycopyrrolate / Formoterol | Aclidinium / Formoterol | 0.86 (0.57-1.34) | Low*,‡ |
| Glycopyrrolate / Indacaterol | Aclidinium / Formoterol | 0.87 (0.63-1.25) | Low*,‡ |
| Tiotropium / Salmeterol | Aclidinium / Formoterol | 1.02 (0.58-1.85) | Low*,‡ |
| Glycopyrrolate / Formoterol | Umeclidinium / Vilanterol | 0.92 (0.60-1.58) | Low*,‡ |
| Glycopyrrolate / Indacaterol | Umeclidinium / Vilanterol | 0.94 (0.72-1.39) | Low *,‡ |
| Tiotropium / Salmeterol | Umeclidinium / Vilanterol | 1.11 (0.65-2.01) | Moderate‡ |
| Glycopyrrolate / Indacaterol | Glycopyrrolate / Formoterol | 1.02 (0.69-1.50) | Low*,‡ |
| Tiotropium / Salmeterol | Glycopyrrolate / Formoterol | 1.19 (0.64-2.19) | Low*,‡ |
| Tiotropium / Salmeterol | Glycopyrrolate / Indacaterol | 1.17 (0.69-1.97) | Low*,‡ |
| All-cause mortality | | | |
| Aclidinium / Formoterol | Tiotropium / Olodaterol | 1.08 (0.3-4.17) | Low*,‡ |
| Umeclidinium / Vilanterol | Tiotropium / Olodaterol | 0.95 (0.22-2.39) | Low‡ |
| Glycopyrrolate / Formoterol | Tiotropium / Olodaterol | 1.18 (0.24-5.26) | Low*,‡ |
| Glycopyrrolate / Indacaterol | Tiotropium / Olodaterol | 0.98 (0.42-2.23) | Low *,‡ |
| Tiotropium / Salmeterol | Tiotropium / Olodaterol | 1.13 (0.26-4.55) | Moderate,‡ |
| Umeclidinium / Vilanterol | Aclidinium / Formoterol | 0.85 (0.15-3.5) | Low*,‡ |
| Glycopyrrolate / Formoterol | Aclidinium / Formoterol | 1.08 (0.16-6.7) | Low*,‡ |
| Glycopyrrolate / Indacaterol | Aclidinium / Formoterol | 0.9 (0.23-3.27) | Low*,‡ |
| Tiotropium / Salmeterol | Aclidinium / Formoterol | 1.03 (0.16-6.14) | Low*,‡ |
| Glycopyrrolate / Formoterol | Umeclidinium / Vilanterol | 1.27 (0.24-8.8) | Low*,‡ |
| Glycopyrrolate / Indacaterol | Umeclidinium / Vilanterol | 1.03 (0.51-3.65) | Low *,‡ |
| Tiotropium / Salmeterol | Umeclidinium / Vilanterol | 1.21 (0.3-6.91) | Moderate‡ |
| Glycopyrrolate / Indacaterol | Glycopyrrolate / Formoterol | 0.83 (0.18-4.16) | Low*,‡ |
| Tiotropium / Salmeterol | Glycopyrrolate / Formoterol | 0.95 (0.13-6.63) | Low*,‡ |
| Tiotropium / Salmeterol | Glycopyrrolate / Indacaterol | 1.16 (0.28-4.33) | Low*,‡ |

* Risk of bias. † Inconsistency. ‡ Indirectness (because of questionable comparability or intransitivity). § Imprecision (§§ Severe imprecision)
