# Supplementary material for: Comparisons of exacerbations and mortality among LAMA/LABA combinations in stable chronic obstructive pulmonary disease: systematic review and Bayesian network meta-analysis
Source: Respir Res. 2020 Nov 25;21:310. doi: 10.1186/s12931-020-01540-8 (PMC7687787; doi:10.1186/s12931-020-01540-8)
Supplement: Supplementary file 7 — Additional file 7. Results of Bayesian network meta-analyses for total exacerbation and all-cause mortality among LAMA and LABA combinations in the network (B). [file 12931_2020_1540_MOESM7_ESM.docx]

**Additional file 7. Results of Bayesian network meta-analyses for total exacerbation and all-cause mortality among LAMA and LABA combinations in the network (B)**

|  | Tiotropium / Olodaterol | Aclidinium / Formoterol | Umeclidinium / Vilanterol | Glycopyrrolate / Formoterol | Glycopyrrolate / Indacaterol | Tiotropium / Salmeterol |
| --- | --- | --- | --- | --- | --- | --- |
| Total exacerbation (16 studies, 39,065 patients) | | | | | | |
| Rank | 3 | 6 | 4 | 1 | 2 | 5 |
| SUCRA | 0.6476 | 0.3322 | 0.4966 | 0.6713 | 0.6572 | 0.3484 |
| NMA estimate OR (95% CrI) |  |  |  |  |  |  |
| LAMA |  |  |  |  |  |  |
| Tiotropium / Olodaterol | 1 |  |  |  |  |  |
| Aclidinium / Formoterol | 1.14 (0.79-1.62) | 1 |  |  |  |  |
| Umeclidinium / Vilanterol | 1.06 (0.68-1.5) | 0.92 (0.58-1.39) | 1 |  |  |  |
| Glycopyrrolate / Formoterol | 0.98 (0.67-1.45) | 0.86 (0.57-1.34) | 0.92 (0.6-1.58) | 1 |  |  |
| Glycopyrrolate / Indacaterol | 1 (0.76-1.34) | 0.87 (0.63-1.25) | 0.94 (0.72-1.39) | 1.02 (0.69-1.5) | 1 |  |
| Tiotropium / Salmeterol | 1.17 (0.67-2.02) | 1.02 (0.58-1.85) | 1.11 (0.65-2.01) | 1.19 (0.64-2.19) | 1.17 (0.69-1.97) | 1 |
| All-cause mortality (16 studies, 39,065 patients) | | | | | | |
| Rank | 3 | 4 | 1 | 6 | 2 | 5 |
| SUCRA | 0.4602 | 0.4187 | 0.4979 | 0.3883 | 0.4675 | 0.3999 |
| NMA estimate OR (95% CrI) |  |  |  |  |  |  |
| LAMA |  |  |  |  |  |  |
| Tiotropium / Olodaterol | 1 |  |  |  |  |  |
| Aclidinium / Formoterol | 1.08 (0.3-4.17) | 1 |  |  |  |  |
| Umeclidinium / Vilanterol | 0.95 (0.22-2.39) | 0.85 (0.15-3.5) | 1 |  |  |  |
| Glycopyrrolate / Formoterol | 1.18 (0.24-5.26) | 1.08 (0.16-6.7) | 1.27 (0.24-8.8) | 1 |  |  |
| Glycopyrrolate / Indacaterol | 0.98 (0.42-2.23) | 0.9 (0.23-3.27) | 1.03 (0.51-3.65) | 0.83 (0.18-4.16) | 1 |  |
| Tiotropium / Salmeterol | 1.13 (0.26-4.55) | 1.03 (0.16-6.14) | 1.21 (0.3-6.91) | 0.95 (0.13-6.63) | 1.16 (0.28-4.33) | 1 |

CrI: credible interval, NMA: network meta-analysis, OR: odds ratio

Median odds ratio with 95% credible interval was calculated as a row to column ratio. If the OR is significantly lower than 1, the drug in the left row is more beneficial than the other drug in the upper column.

* indicates that the posterior probability is either less than 0.025 or more than 0.975, which is considered statistically significant.
