# Supplementary material for: Comparisons of exacerbations and mortality among LAMA/LABA combinations in stable chronic obstructive pulmonary disease: systematic review and Bayesian network meta-analysis
Source: Respir Res. 2020 Nov 25;21:310. doi: 10.1186/s12931-020-01540-8 (PMC7687787; doi:10.1186/s12931-020-01540-8)
Supplement: Supplementary file 8 — Additional file 8. Consistency evaluation between Bayesian network meta-analyses and direct meta-analysis. [file 12931_2020_1540_MOESM8_ESM.docx]

**Additional file 8. Consistency between the results from Bayesian network meta-analyses and direct meta-analysis**

| Treatment | Comparator | Network meta-analysis estimate OR | | Posterior Probability (OR>1) | Direct meta-analysis estimate OR | | Consistency assumption |
| --- | --- | --- | --- | --- | --- | --- | --- |
|  |  | **Posterior median** | **95% CrIs** |  | **OR** | **95% CIs** | **P-value** |
| Total exacerbation | | | | | | | |
| Tiotropium / Olodaterol | Tiotropium | 0.97 | 0.78-1.22 | 0.35 | 0.96 | 0.82-1.12 | 0.16 |
| Aclidinium / Formoterol | Tiotropium | 0.96 | 0.62-1.47 | 0.41 | - | - | - |
| Umeclidinium / Vilanterol | Tiotropium | 0.41 | 0.19-0.93 | 0.02 | - | - | - |
| Glycopyrrolate / Formoterol | Tiotropium | 0.9 | 0.63-1.28 | 0.24 | 0.89 | 0.69-1.15 | 0.50 |
| Glycopyrrolate / Indacaterol | Tiotropium | 1 | 0.78-1.3 | 0.49 | 0.99 | 0.81-1.22 | 0.53 |
| Tiotropium / Salmeterol | Tiotropium | 1.09 | 0.63-1.92 | 0.63 | 1.09 | 0.68-1.75 | 0.50 |
| Moderate to severe exacerbation | | | | | | | |
| Tiotropium / Olodaterol | Tiotropium | 1.03 | 0.34-3 | 0.59 | 1.03 | 0.95-1.13 | 0.50 |
| Aclidinium / Formoterol | Tiotropium | 0.98 | 0.28-3.35 | 0.48 | - | - | - |
| Umeclidinium / Vilanterol | Tiotropium | 1.07 | 0-82530 | 0.5 | - | - | - |
| Glycopyrrolate / Formoterol | Tiotropium | 0.94 | 0.36-2.49 | 0.38 | 0.92 | 0.71-1.20 | 0.50 |
| Glycopyrrolate / Indacaterol | Tiotropium | 1.11 | 0.44-2.93 | 0.71 | 1.13 | 0.92-1.38 | 0.50 |
| Tiotropium / Salmeterol | Tiotropium | - | - | - | - | - | - |
| All-cause mortality | | | | | | | |
| Tiotropium / Olodaterol | Tiotropium | 0.97 | 0.25-4 | 0.46 | 0.93 | 0.73-1.17 | 0.57 |
| Aclidinium / Formoterol | Tiotropium | 0.88 | 0.07-15.28 | 0.46 | - | - | - |
| Umeclidinium / Vilanterol | Tiotropium | 0.02 | 0-5.96 | 0.09 | - | - | - |
| Glycopyrrolate / Formoterol | Tiotropium | 0.71 | 0.07-11.08 | 0.37 | 0.35 | 0.09-1.29 | 0.50 |
| Glycopyrrolate / Indacaterol | Tiotropium | 1.03 | 0.3-4.67 | 0.52 | 1.00 | 0.59-1.72 | 0.37 |
| Tiotropium / Salmeterol | Tiotropium | 1.64 | 0.15-18.89 | 0.69 | 1.61 | 0.44-5.81 | 0.50 |
| COPD-related mortality | | | | | | | |
| Tiotropium / Olodaterol | Tiotropium | 0.69 | 0.05-12.58 | 0.31 | 0.67 | 0.44-1.03 | 0.36 |
| Aclidinium / Formoterol | Tiotropium | 0.01 | 0-880 | 0.20 | - | - | - |
| Umeclidinium / Vilanterol | Tiotropium | 1.37 | 0-134800 | 0.52 | - | - | - |
| Glycopyrrolate / Formoterol | Tiotropium | 0.04 | 0-41900 | 0.34 | 0.44 | 0.01-21.96 | 0.41 |
| Glycopyrrolate / Indacaterol | Tiotropium | 29.96 | 0-5780000 | 0.71 | - | - | - |
| Tiotropium / Salmeterol | Tiotropium | - | - | - | - | - | - |
| Cardiovascular disease-related mortality | | | | | | | |
| Tiotropium / Olodaterol | Tiotropium | 1.21 | 0.08-23.2 | 0.58 | 1.17 | 0.39-3.48 | 0.50 |
| Aclidinium / Formoterol | Tiotropium | 0.05 | 0-24.05 | 0.17 | - | - | - |
| Umeclidinium / Vilanterol | Tiotropium | 0.42 | 0-10890 | 0.43 | - | - | - |
| Glycopyrrolate / Formoterol | Tiotropium | 1.71 | 0.05-147.5 | 0.62 | 1.15 | 0.10-12.72 | 0.50 |
| Glycopyrrolate / Indacaterol | Tiotropium | 1.51 | 0-1020000 | 0.53 | - | - | - |
| Tiotropium / Salmeterol | Tiotropium | - | - | - | - | - | - |
| Major adverse cardiac events | | | | | | | |
| Tiotropium / Olodaterol | Tiotropium | 0.92 | 0.09-10.01 | 0.43 | 0.92 | 0.67-1.25 | 0.50 |
| Aclidinium / Formoterol | Tiotropium | 0.22 | 0.01-3.59 | 0.11 | - | - | - |
| Umeclidinium / Vilanterol | Tiotropium | - | - | - | - | - | - |
| Glycopyrrolate / Formoterol | Tiotropium | 0.37 | 0.04-3.81 | 0.13 | 0.38 | 0.15-1.00 | 0.50 |
| Glycopyrrolate / Indacaterol | Tiotropium | 1.8 | 0.21-29.42 | 0.72 | 1.33 | 0.30-5.98 | 0.03 |
| Tiotropium / Salmeterol | Tiotropium | - | - | - | - | - | - |
| Pneumonia | | | | | | | |
| Tiotropium / Olodaterol | Tiotropium | 1.05 | 0.48-2.22 | 0.6 | 1.07 | 0.79-1.46 | 0.82 |
| Aclidinium / Formoterol | Tiotropium | 0.89 | 0.16-4.77 | 0.44 | - | - | - |
| Umeclidinium / Vilanterol | Tiotropium | 0.69 | 0.04-10.71 | 0.38 | - | - | - |
| Glycopyrrolate / Formoterol | Tiotropium | 1.34 | 0.4-4.46 | 0.74 | 1.91 | 0.78-4.67 | 0.50 |
| Glycopyrrolate / Indacaterol | Tiotropium | 1.24 | 0.66-3.35 | 0.76 | 1.01 | 0.65-1.57 | <0.01 |
| Tiotropium / Salmeterol | Tiotropium | 25.94 | 0.14-167300 | 0.88 | 3.18 | 0.13-78.75 | 0.68 |

CrI: credible interval, ICS: inhaled corticosteroid, LABA: long-acting beta-agonist, LAMA: long-acting muscarinic antagonist, NMA: network meta-analysis, OR: odds ratio, Sd: standard deviation

^a^ Effects of ICS on MACE compared to other drug classes or placebo were not analyzed because there were no studies involving ICS.
